# Deficiency in circulating T cells in four core genotypes mice with *Sry* translocation

**DOI:** 10.1093/jimmun/vkag207

**Published:** 2026-08-13

**Authors:** Kaitlin E McKernan, Jacqueline-Yvonne Cephus, Shelby N Kuehnle, Emely Henriquez-Pilier, Aamina C Dandy, Tess Bacharier, Vasiliy V Polosukhin, Katherine N Cahill, Patricia Silveyra, Dawn C Newcomb

**Affiliations:** Department of Pathology, Microbiology, and Immunology, Vanderbilt University Medical Center, Nashville, TN, United States; Vanderbilt Center for Immunobiology, Vanderbilt University Medical Center, Nashville, TN, United States; Department of Medicine, Vanderbilt University Medical Center, Nashville, TN, United States; Department of Medicine, Vanderbilt University Medical Center, Nashville, TN, United States; Department of Pathology, Microbiology, and Immunology, Vanderbilt University Medical Center, Nashville, TN, United States; Vanderbilt Center for Immunobiology, Vanderbilt University Medical Center, Nashville, TN, United States; Department of Pathology, Microbiology, and Immunology, Vanderbilt University Medical Center, Nashville, TN, United States; Vanderbilt Center for Immunobiology, Vanderbilt University Medical Center, Nashville, TN, United States; Department of Medicine, Vanderbilt University Medical Center, Nashville, TN, United States; Department of Medicine, Vanderbilt University Medical Center, Nashville, TN, United States; Department of Medicine, Vanderbilt University Medical Center, Nashville, TN, United States; Department of Environmental and Occupational Health, Indiana University School of Public Health-Bloomington, Bloomington, IN, United States; Department of Pathology, Microbiology, and Immunology, Vanderbilt University Medical Center, Nashville, TN, United States; Vanderbilt Center for Immunobiology, Vanderbilt University Medical Center, Nashville, TN, United States; Department of Medicine, Vanderbilt University Medical Center, Nashville, TN, United States

**Keywords:** CD4^+^ T Cells, CD8^+^ T cells, four core genotypes (FCG) mouse model

## Abstract

Sex differences exist in the immune responses to infections and in the prevalence and severity of autoimmune and allergic diseases. These sex differences may be caused by sex hormones and/or variable inactivation of X chromosome genes. The 4 core genotypes mice allow for distinction between the effects of sex hormones and chromosomes on physiology and disease pathology. In the FCG mouse model, the *Sry* gene is deleted from the Y chromosome and inserted into chromosome 3 as multiple copies of a transgene, allowing for phenotypic male and female mice with XX and XY chromosomes. We sought to investigate the role of sex hormones and chromosomes in respiratory syncytial virus infection and allergen-induced airway inflammation. However, in performing these studies, we found that the immune response in FCG males (XXM and XYM) was significantly blunted. XXF and XYF had 4-fold more CD3^+^CD4^+^ T cells and over 7-fold more CD3^+^CD8^+^ T cells compared to XXM and XYM in the lungs following stimulus. CD4^+^ and CD8^+^ T cells were also significantly decreased in XXM and XYM mice in the lungs, spleen, and peripheral blood at baseline with no effect on B cells, NK cells, or myeloid cells. Thymic T cell numbers were similar among groups, and bone marrow progenitors were unchanged between groups. Overall, the translocation of *Sry* to chromosome 3 resulted in dramatically decreased immune responses to RSV infection and allergen-challenge, indicating that FCG male mice do not mount appropriate immune responses to a respiratory virus infection.

## Introduction

Sex differences exist in the immune responses to acute infections as well as the prevalence of inflammatory diseases. Women have an increased prevalence of autoimmune diseases, including lupus and multiple sclerosis,^1^^,^^2^ as well as an increased prevalence of asthma and allergies.^3–9^ Additionally, women have a stronger immune response to pathogens, resulting in diminished incidence and severity of many bacterial and viral infections compared to men.^10–18^ The sex bias in immune response also exists in childhood, with boys having increased respiratory viral complications, increased allergic disease, and increased rates of asthma compared to girls.^3^^,^^8^^,^^9^^,^^19–23^ Our previous works showed that ovarian hormones increased and that androgens decreased allergen-induced airway inflammation in mouse models of asthma,^24–28^ but the mechanisms underlying the sex difference in childhood asthma, when sex hormones are low, remain unclear.

Sex differences impacting inflammatory pathways in the immune response may be caused by sex hormones and/or variable inactivation of genes on the X chromosomes. Androgens decreased neutrophil and CD8^+^ T cell killing, CD4^+^ T cell effector function, B cell frequency, and antibody responses,^29^^,^^30^ and type 2 innate lymphoid cell (ILC2) function, and increased T regulatory (Treg) cell suppressive function.^25–27^^,^^31–33^ In contrast, estrogen has a dose-dependent effect, with lower physiologic doses increasing immune function and higher doses present during pregnancy decreasing immune function.^34^^,^^35^ Estrogen signaling through estrogen receptor alpha increased IL-33 release from airway epithelial cells, increased CD4^+^ Th17 cell proliferation, and increased CD4^+^ T cell metabolism.^24^^,^^28^^,^^36^^,^^37^ Sex differences in inflammatory pathways and immune-related diseases may also be driven by sex chromosome dosage differences, since the X chromosome is rich in immune-related genes, including *Foxp3*, *Tlr7*, *Tlr8*, and *Cxcr3.*^38–40^ In females, X inactivation of one gene occurs to prevent both X chromosomes from expressing genes. However, 15%–30% of human X-linked genes^41–43^ and 3%–7% of genes in mice^44^^,^^45^ escape X inactivation, resulting in increased expression of these genes in females compared to males.

To untangle the contribution of sex hormones and sex chromosomes in allergen-induced airway inflammation, we utilized the four core genotypes (FCG) mice where the *Sry* gene is deleted from the Y chromosome and inserted into chromosome 3 as multiple copies of a transgene. Translocation of the *Sry* gene onto an autosome (chromosome 3) provides the ability to isolate the impact of sex chromosomes, and X-linked inactivation, versus sex hormones on diseases.^46^ However, we confirmed prior studies showing that the translocation of *Sry* to chromosome 3 dramatically decreased CD4^+^ and CD8^+^ T cells in peripheral tissues and in systemic circulation with no effect on B cells or myeloid cells. Additionally, we determined that *Sry* translocation to chromosome 3 resulted in no T cell–mediated response to respiratory syncytial virus (RSV) infection, preventing clearance of the virus at 7 d.

## Materials and methods

### Mice

All experiments were performed at Vanderbilt University in accordance with Institutional Animal Care and Utilization Committee (IACUC)-approved protocols (M230009-00) and conformed to all relevant regulatory standards. All mice were originally purchased from Jackson Laboratories (Bar Harbor, Maine, USA). Mice were housed in a pathogen-free facility with up to 5 mice per cage and ad libitum access to food and water. This study used an inbred strain of the FCG mouse model (strain no. 010905). C57BL/6J wild-type females (C57BL/6J) were bred with the XYM male mice to produce pups belonging to one of the FCG (XXM, XXF, XYM, and XYF) and C57BL/6J wild-type males (WTM) were used as controls (strain no. 000664). Mice were genotyped after weaning using Transnetyx. Mice were utilized as described below. Adult mice were 10–14 wk old at the start of experiments and were housed as littermates. For all experiments described below, mice were euthanized with a pentobarbital overdose.

### Generation of respiratory syncytial virus (RSV) stock

HEp-2 cells were obtained from ATCC. HEp-2 cells were cultured in minimal essential media (MEM) supplemented with 10% fetal bovine serum (FBS) R&D Systems, 1% penicillin/streptomycin (Gibco) and 0.5 mL gentamicin (Life Technologies 15710064). RSV strain 01-2/20 was isolated in 2001 from the Vanderbilt Vaccine Clinic (Nashville, Tennessee, USA).^47^ RSV was propagated and titrated in HEp-2 cells as previously described.^48^ Briefly, master RSV stock was utilized to infect HEp-2 cells in flasks for 1 h, and cells were cultured until 70% cytopathic effect (CPE) developed. Cells were then sonicated, and culture supernatants were collected as RSV working stocks. Viral titers were determined by plaque assay with serial dilutions of RSV in HEp-2 cells. Mock inoculum was prepared by collected cell culture supernatant from lysed, uninfected HEp-2 cells.

### RSV infection

Mice were anesthetized with isoflurane and infected intranasally with 01/2-20 RSV, 0.8 × and 0.25 × 10^6^ PFU/neonatal mouse, or mock inoculum. Weight loss was measured every other day. Mice were sacrificed 2, 4, or 7 d post infection (dpi) for endpoint analysis.

### House dust mite (HDM) allergen exposure

Mice were anesthetized with isoflurane and sensitized intranasally with 25 μg/80 μL HDM (*Dermatophagoides pteronyssinus*) (Greer Lot no. 406945) or PBS on days 0 and 3 and challenged with 25 μg/80 μL HDM or PBS on days 14–17. Twenty-four hours after the last challenge, mice were sacrificed for endpoint analysis.

### Bronchoalveolar lavage (BAL) collection

Immediately following euthanasia, BAL fluid was obtained through the insertion of a tracheostomy tube, instillation of 800 µL of PBS through a syringe into the lungs, gentle massage of the lungs, and gentle withdrawal of fluid through the same syringe. Total cells were determined, and BAL cell suspension were adhered to a slide and stained using a commercially available Three-Step Stain kit (Richard-Allen Science, Thermo Fisher Scientific).^25^ Using light microscopy, BAL cells were classified as eosinophils, neutrophils, macrophages/monocytes, or lymphocytes. The percentage of each cell type was determined to calculate the total number.

### RNA isolation and quantitative real time PCR analysis

Total RNA from lungs and thymus was isolated using the RNeasy Mini Kit (Qiagen, Hilden, Germany). Complementary (cDNA) was generated using 250–500 ng of total RNA (standardized per batch). TaqMan quantitative PCR (qPCR) analysis of and tata box protein (*Tbp*) (Thermo Fisher Scientific Hs00427620_m1 4331182) and SYBR Green qPCR analysis of RSV M protein mRNA (Integrated DNA Technologies [IDT] RSV M Protein Forward Sequence 5'-GGC AAA TAT GGA AAC ATA CGT GAA-3' cat no. 433539861 and RSV M Protein Reverse Primer Sequence 5'-TCT TTT TCT AGG ACA TTG TAY TGA ACA-3' cat no. 433539862) expression was conducted using commercially available primers and FAM/MGB probes. Data were reported as relative expression normalized to the housekeeping gene *Tbp*.

### Cytokine and antibody measurements

Left lungs were snap-frozen in liquid nitrogen at the time of harvest. Lungs were mechanically disrupted using 1 mL of MEM media and homogenized via BeadBeater (BioSpec Products, Bartletsville, Oklahoma, USA). Lung cytokine measurements (IFN-γ, IL-13, IL-17A) were performed using Quantikine enzyme-linked immunosorbent assay (ELISA) kits according to manufacturer instructions (R&D Systems, Minneapolis, Minnesota, USA).

### Cell counting

Cells were counted by staining with 0.4% Trypan Blue Solution (Gibco, Grand Island, New York, USA) and manually counting under the microscope utilizing a hemocytometer. Counts were averaged across 4 panels (× 10^4^) as indicated by hemocytometer manufacturing instructions.

### Generating immune single cell suspensions from tissues

After sacrifice, multiple tissues and peripheral blood were collected from mice. Lungs and whole thymuses were harvested and digested using 1 mg/mL collagenase type IV and DNase I in RPMI with 5% FBS for 30 min and 37 °C. Digestion was stopped using 1 µM EDTA. Cells were filtered through a 70 µm strainer to remove debris using RPMI with 5% FBS and a red blood cell (RBC) lysis (BioLegend, San Diego, California, USA) was performed according to the manufacturer’s instructions with 5 mL RBC Lysis Buffer and washed. Spleens were harvested and filtered through a 70 µm strainer using RPMI with 5% FBS to remove debris and an RBC lysis (BioLegend, San Diego, California, USA) was performed according to the manufacturer’s instructions and washed. For bone marrow, femurs and tibias were collected and skin and muscles removed. Clean bones were cut on one end and centrifuged at 5,000 rpm for 2 min to collect bone marrow cells. Cells then underwent RBC Lysis (BioLegend, San Diego, California, USA) according to manufacturer’s instructions at 4 °C for 5 min and washed. For all tissues, cells were counted following RBC lysis step.

### Peripheral blood collection

Around 100–200 μL whole blood was collected via retro-orbital injections immediately following anesthesia via ketamine/xylazine into EDTA-coated tubes (SAI Technologies cat no. PMTP-S-1.3) prior to sacrifice. RBC lysis was performed by adding 1 mL RBC buffer (BioLegend, San Diego, California, USA) and incubating at 4 °C for 5 min. The reaction was then quenched with 5–10 mL PBS + 3% FBS and cells were pelleted at 2,000 rpm for 5 min. RBC lysis, quenching, and pelleting was repeated to remove remaining RBCs. Cells were then washed with PBS and counted.

### Flow cytometry

Single cell suspensions from lungs, thymus, spleen, bone marrow, or peripheral blood were obtained as described above. Cells were stained with fixable viability dye Live Dead Aqua (Thermo Fisher Scientific, Waltham, Massachusetts, USA) for 20 min and washed with PBS with 3% FBS. Cells were then incubated with 1:1000 anti-FcR (CD16 and CD32) antibody (BioLegend cat no. 101319, San Diego, California, USA) to prevent non-specific staining followed by staining for surface markers (Table S1) at 4 °C in the dark for 45 min. Cells were washed with PBS plus 3% FBS and then fixed and permeabilized using the FoxP3 Transcription Factor Fix/Perm Kit (eBioscience, San Diego, California, USA) at 4 °C in the dark for 20 min. Cells were washed 2× with Perm Wash and resuspended in PBS with 3% FBS. Flow cytometry was conducted on a Cytek Aurora and data analyzed using FlowJo.

In select experiments, lung single cell suspensions were restimulated in IMDM with GlutaMax and HEPES, 10% FBS, 1% penicillin/streptomycin, 1% sodium pyruvate, 50 µM 2-mercaptoethanol, 1 µM ionomycin, 50 ng/mL PMA, and 0.07% Golgi Stop at 37 °C for 4 h. Cells were then washed with PBS and surface stained for flow cytometry as described above. After the cells were fixed and permeabilized, cells were washed with Perm Wash and stained for intracellular markers (Table S2) at 4 °C in the dark for 45 min. Cells were washed 2× with Perm Wash and resuspended in PBS with 3% FBS. Flow cytometry was conducted on a Cytek Aurora and data analyzed using FlowJo (BD Biosciences) with mean fluorescence intensity (MFI) calculated using the geometric mean.

### Histopathology

To measure viral-induced airway inflammation, mice were euthanized 7 dpi. Lungs were perfused, inflated, and instilled with 800 μL neutral-buffered formalin overnight at room temperature. Lungs were transferred to 70% ethanol and paraffin embedded. Tissue section (4 μm) were stained with hematoxylin and eosin (H&E). Slides were quantified and scored according to following scale by a pathologist blinded to the experimental groups: 0 indicated no inflammatory cells; 1, a few inflammatory cells; 2, increased accumulation of inflammatory cells; and 3, abundant accumulation of inflammatory cells.

### Statistical analysis

Statistical analyses were performed using GraphPad Prism (v. 10) with data represented as mean ± SEM as denoted in the legend. Data were significant when *P* was less than 0.05. Comparisons between 2 groups were performed using 2-way *t* test or Mann–Whiteny *U* test for parametric or nonparametric data, respectively. Comparisons of greater-than 2 groups were made using an ANOVA with post hoc for multiple comparisons.

## Results

### XXM and XYM four core genotypes (FCG) mice have a deficit in lung CD3^+^CD4^+^ and CD3^+^CD8^+^ T cells in the lung following respiratory syncytial virus (RSV) infection

**Figure 1:**
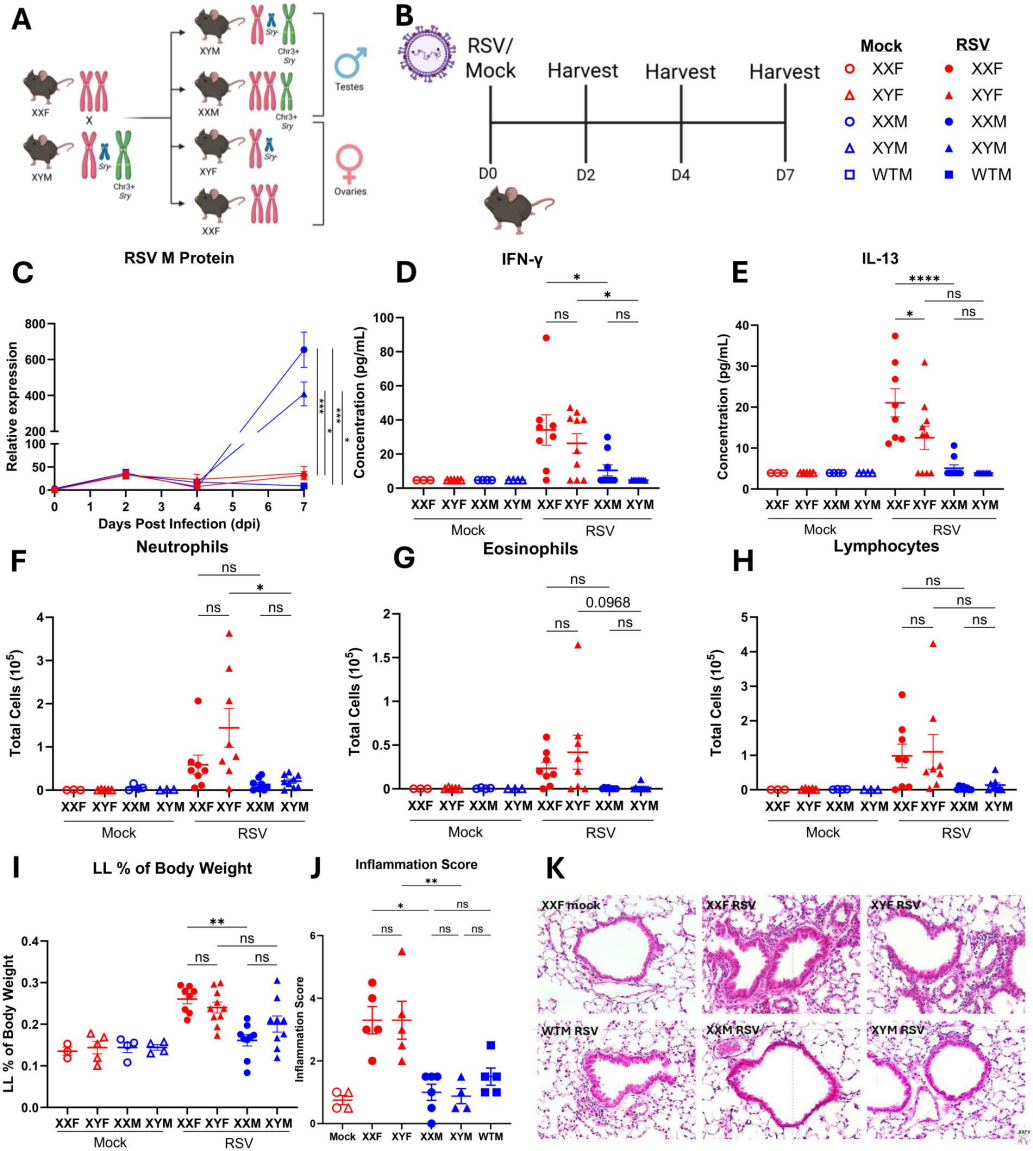
XXM and XYM FCG mice have a decreased immune response in the lungs following RSV infection. (A) FCG model breeding scheme with genotypes and phenotypes. (B) Model of RSV infection. Adult XX female (XXF), XYF, XX male (XXM), and XYM FCG mice were infected intranasally with RSV 01-2/20 or mock (HEp2 cells). Lungs were harvested 2-, 4-, and 7-days post infection (dpi) and BAL fluid was harvested 4 and 7 dpi. (C) Time course of RSV M protein expression in the lungs 2, 4, and 7 dpi. Mock samples served as day 0 post infection. (D–E) IFN-γ and IL-13 protein expression in lung homogenates 7 dpi. (F–H) Quantification of neutrophils, eosinophils, and lymphocytes in the BAL fluid 7 dpi. (I) Left lung % of total body weight at 7 dpi. (J) Quantification of inflammation in the lung 7 dpi. (K) Representative H&E-stained sections in at 20X magnification 7 dpi. Data are expressed as mean ± SEM: *n* = 3–9 mice per group combined from 2 independent experiments. Two-way ANOVA with Tukey’s post hoc. * *P* < 0.05, **** *P* < 0.0001. Graphical images (A and B) were created with BioRender.com.

A colony of 4 core genotypes (FCG) mice was created by breeding XY male (XYM) FCG mice to WT female C57BL/6J mice. XYM mice have the *Sry* gene deleted from the Y chromosome and an *Sry* transgene inserted on chromosome 3. This breeding strategy results in four distinct genotypes: XX *Sry*^−^ phenotypic females (XXF), XY *Sry*^−^ phenotypic females (XYF), XX *Sry*^+^ phenotypic males (XXM), and XY *Sry*^+^ phenotypic males (XYM) (Fig. 1A). To investigate the role of sex hormones versus sex chromosomes in RSV-induced airway inflammation, adult XXF, XYF, XXM, and XYM FCG mice were infected with RSV 01-2/20, a mucogenic strain known to induce a mixed Th1 and Th2 immune response,^48^ or mock inoculum as shown in Fig. 1B. As a proxy for RSV infection kinetics, RSV M gene expression was determined 2, 4, and 7 dpi. To compare viral titers between groups, lungs were harvested from infected and mock infected FCG XXF, XYF, XXM, and XYM and WT male mice 2, 4, and 7 dpi and RSV M protein gene expression was assessed by qPCR. Mice were successfully infected and there were no differences in viral titer between groups on day 2 and day 4 post infection (Fig. 1C). There were also no differences in total BAL cells, macrophages, neutrophils, lymphocytes, or eosinophils (Fig. S4A) at 4 dpi between any of the groups. However, there was a significant increase in RSV M protein gene expression in XXM and XYM compared to XXF, XYF, and WTM at 7 dpi (Fig. 1C). Whole lungs and bronchoalveolar lavage (BAL) fluid were also collected 7 dpi. Lung IFN-γ protein levels were significantly increased in XXF and XYF compared to XXM and XYM, respectively, and these cytokines were not detected in mock samples (Fig. 1D). Lung IL-13 was also significantly increased in XXF following infection (Fig. 1E). IL-17A was also measured and was not detected in any of the samples (data not shown). After RSV infection, a significant increase in neutrophils was detected in XYF compared to XYM (Fig. 1F). No differences in total BAL cells, macrophages, eosinophils, and lymphocytes were detected between any of the RSV infected groups (Fig. S1A, B and Fig. 1G, H). Given that XXF and XYF had increased lung inflammatory cytokine levels and BAL neutrophils and that XXM and XYM had increased lung viral titer 7 dpi, we next asked whether there were differences in overall lung edema. Compared to mock samples, XXF and XYF had significantly increased lung edema at 7 dpi, as evidence by heavier lung weights as a percent of total body weight (Fig. 1I). XXF had significantly greater lung edema than XXM and there was a trend towards increased edema in XYF compared to XYM mice (Fig. 1I). Because we observed increased edema in the FCG female mice following RSV infection, we then sought to assess lung inflammation by histopathology. XXF and XYF had increased lung inflammation as measured by H&E staining compared to XXM and XYM FCG mice (Fig. 1J, K). We also assed H&E in WTM mice, and they had lung inflammation intermediate to FCG females and FCG males. (Fig. 1J, K). Goblet cell metaplasia measured by PAS staining was low and only present in a few female FCG and WTM samples, and there were no significant differences between all groups (data not shown). Taken all together, these data imply that compared to XXM and XYM, XXF and XYF FCG mice have an increased inflammatory response to RSV infection contributing to increased viral clearance.

**Figure 2:**
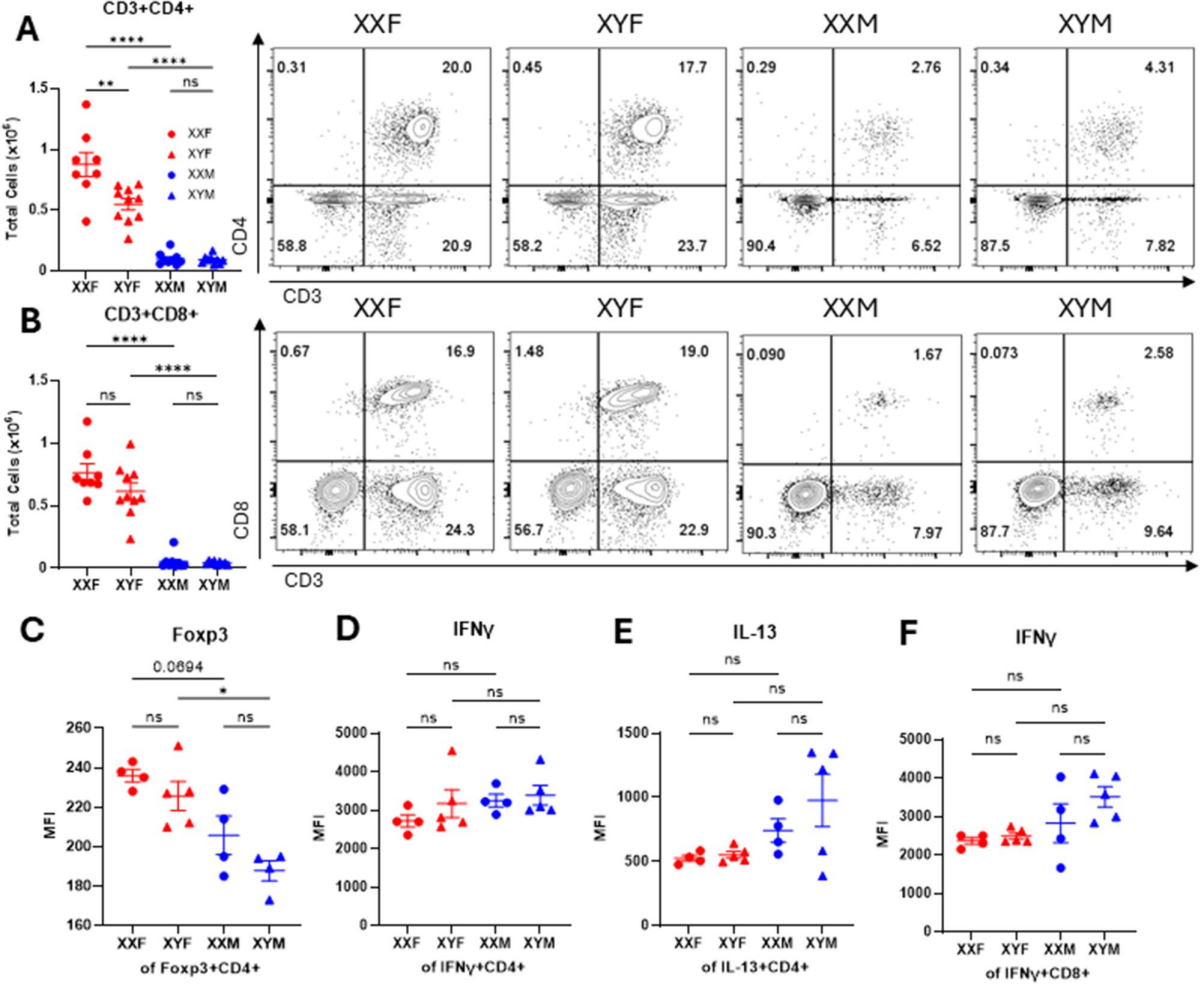
XXM and XYM FCG male mice have a deficit in their CD3^+^CD4^+^ and CD3^+^CD8^+^ Cells 7 dpi RSV infection in the lungs. (A) Quantification and representative flow diagrams of CD3^+^CD4^+^ cells after RSV infection. (B) Quantification and representative flow diagrams of CD3^+^CD8^+^ cells after RSV infection (with all cells pre-gated on viable CD45^+^ cells). (C–E) Mean fluorescent intensity (MFI) expression of Foxp3, IFN-γ, and IL-13 in lung CD3^+^CD4^+^ cells. (F) MFI of IFNγ in lung CD3^+^CD8^+^ cells. Data are expressed as mean ± SEM: *n* = 6–9 mice per group combined from 2 independent experiments in A–B and *n* = 4–5 from 1 independent experiment. Two-way ANOVA with Tukey’s post hoc. * *P* < 0.05, ** *P* < 0.01, *** *P* < 0.001, *P* < 0.0001.

We next examined whether sex differences by sex hormones or sex chromosomes existed in CD4^+^ or CD8^+^ T cells or NK cells in the lungs 7 d after RSV infection. Total CD4^+^ and CD8^+^ T cells, Tregs (Foxp3^+^CD4^+^), Th1 cells (IFN-γ^+^CD4^+^), Th2 cells (IL-13^+^CD4^+^), Th17 cells (IL-17A^+^CD4^+^), effector CD8^+^ T cells (IFN-γ^+^CD8^+^), and NK cells (CD3-NKp46^+^) were determined by flow cytometry using the gating strategy shown in Fig. S2. Strikingly, XXF and XYF had about 4-fold more CD3^+^CD4^+^ T cells and over 7-fold more CD3^+^CD8^+^ T cells compared to XXM and XYM in the lungs following infection (Fig. 2A, B). Concordant increases in total lung Tregs, Th1, Th2, and IFN-γ^+^CD8^+^ T cells were seen in XXF and XYF mice compared to XXM and XYM mice, but there was no difference in total Th17 cells (Fig. S3A, B). Interestingly, there were no differences in total TCR-γδ T cells between the groups, and there was a slight increase in total NK cells and IFN-γ^+^ NK cells in the XXF mice following infection (Fig. S3B). These trends in T cell numbers were similar at 4 d post infection, with XXF and XYF having a significant increase in CD3^+^CD4^+^ and CD3^+^CD8^+^ T cells compared to XXM and XYM (Fig. S4B, C). WTM mice also had significantly elevated CD3^+^CD4^+^ and CD3^+^CD8^+^ T cells compared to XXM and XYM (Fig. S4B, C). This was also accompanied by a significant increase in total lung Tregs, Th1, and Th2 cells and a trend towards increased IFN-γ^+^CD8^+^ T cells in the XXF and XYF and a trend towards increased Tregs, Th1, and Th2 cells in WTM mice with no difference in total Th17 cells (Fig. S4B, C). There was also no difference in NK cells at 4 dpi between groups (Fig. S4B, C). Given the significant deficit in CD4^+^ and CD8^+^ T cells in the male FCG mice, we next asked whether these cells expressed different levels of cytokines at 7 dpi. Mean fluorescent intensity (MFI) of Foxp3 in CD4^+^ T cells was significantly increased in the XXF and XYF, but IFN-γ MFI and IL-13 MFI in CD4^+^ T cells and IFNγ MFI in CD8^+^ T cells were not different between the groups (Fig. 2C–F). Collectively these data suggest that XXM and XYM mice have a deficit in CD4^+^ and CD8^+^ T cell total numbers, but not in cytokine production from Th1, Th2, and CD8^+^ T cells following RSV infection.

Since it is known that sex hormones affect T cell number and function,^24–27^^,^^31–33^^,^^35^^,^^37^^,^^49^^,^^50^ we sought to determine whether this drastic difference in T cell numbers was present in younger pre-pubertal mice. As sex hormones have been shown to rise around 4.5 wk in mice, we first infected 3-wk-old mice with RSV. Briefly, timed breedings were set up and mice were infected on postnatal day 21 (PND 21) at the time of weaning and lungs harvested 7 dpi (on PND 28) (Fig. S5A). Utilizing flow cytometry, we found a similar deficit in T cells, with XXM and XYM having a 4-fold decrease in CD3^+^CD4^+^ T cells and a 7-fold decrease in CD3^+^CD8^+^ T cells (Fig. S5B). Total Tregs, Th1 cells, and effector IFNγ-^+^CD8^+^ T cells were also significantly decreased in XXM and XYM mice, and Th17 cells were significantly decreased in XXM compared to XXF mice while Th2 cells were significantly decreased in XYM compared to XYF mice (Fig. S5C). We also checked if this difference was also present in younger mice before sex hormones rise. Timed breedings were set up and mice were infected with RSV on postnatal day 7 and lungs harvested 7 dpi (PND 14) (Fig. S5D). In these mice, a T cell deficit also existed, but to a lesser extent, with XXM and XYM CD4^+^ T cells decreased by 3-fold and CD8^+^ T cells decreased by around 4-fold compared to females (Fig. S5E). Total numbers of Th1, Th2, Th17, and IFN-γ^+^CD8^+^ T cells were also decreased in male FCG mice, and there was no difference in Treg numbers between groups (Fig. S5F). Other groups have previously not found any difference in serum testosterone levels between FCG male mice and WT male mice in adults, and anogenital distance did not differ for 21 d after birth, suggesting similar levels of exposure to perinatal testosterone peaks.^51^^,^^52^ Overall, these data demonstrate a deficit in CD4^+^ and CD8^+^ T cell numbers in FCG male mice in the lungs that is present early in life, a phenomenon that is driven by the presence of ovaries versus testes, the expression of *Sry*, and/or sex hormones rather than sex chromosomes.

### XXM and XYM FCG mice have a deficit of CD4^+^ and CD8^+^ T cells in the lungs following Allergen-induced airway inflammation compared to female FCG mice and WT male mice

**Figure 3:**
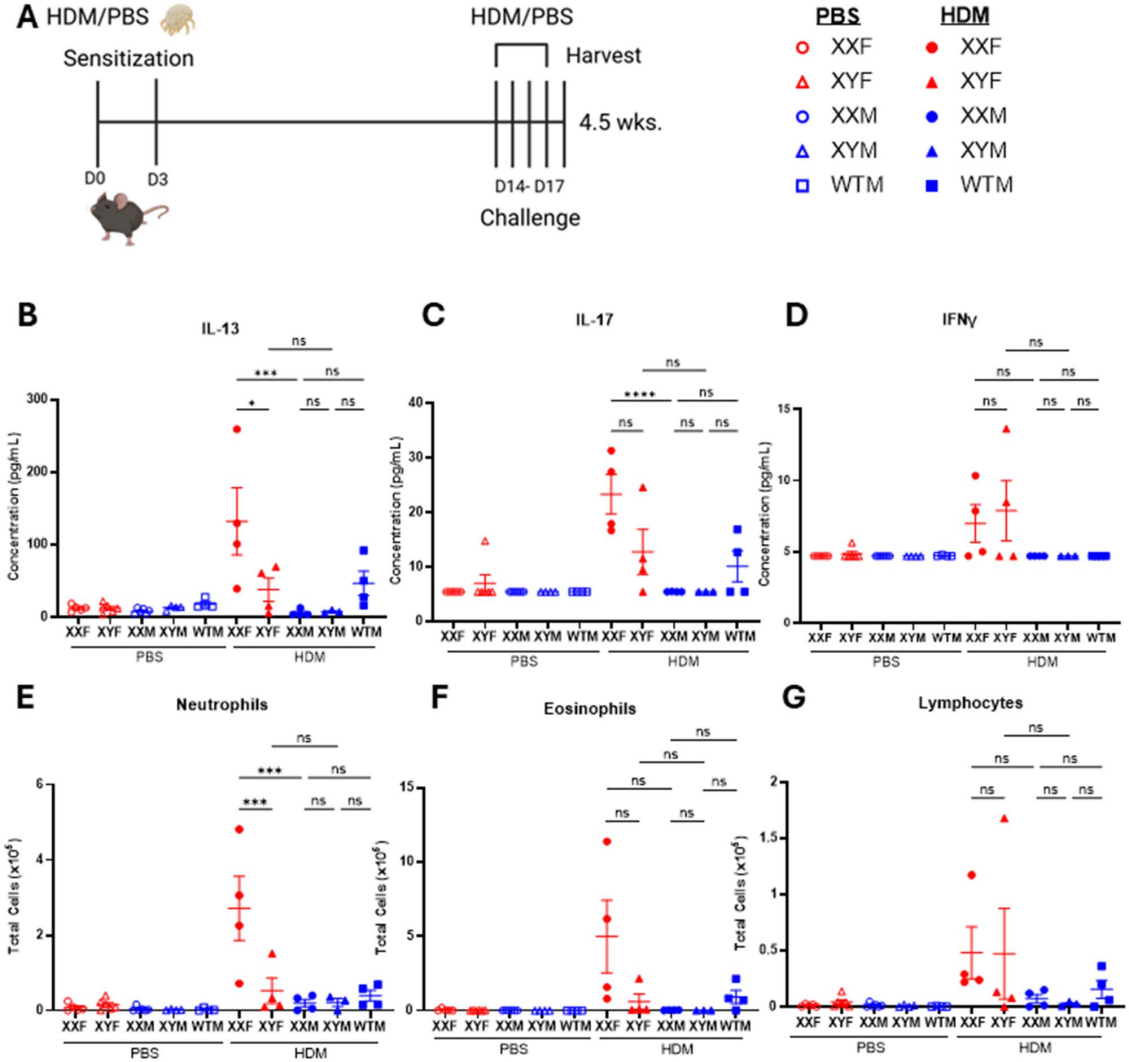
XXM and XYM FCG male mice have a deficit in immune response following house dust mite (HDM)-induced airway inflammation. (A) Model of HDM sensitization and challenge protocol. XXF, XYF, XXM, and XYM FCG mice and WT C57BL/6 male mice were intranasally sensitized with 25 μg of HDM on days 0 and 3 and challenged with 25 μg of HDM on days 14–17. As a control, mice were administered PBS with the same protocol. Serum, BAL fluid, and lungs were harvested 24 h after the last challenge. (B–D) Lung IL-13, IL-17A, and IFN-γ protein expression. (E–G) Quantification of BAL neutrophils, eosinophils, and lymphocytes. Data are expressed as mean ± SEM: *n* = 3–5 mice per group. Two-way ANOVA with Tukey’s post-hoc, * *P* < 0.05, *** *P* < 0.001, **** *P* < 0.0001. Graphical image (A) was created with Biorender.com.

**Figure 4:**
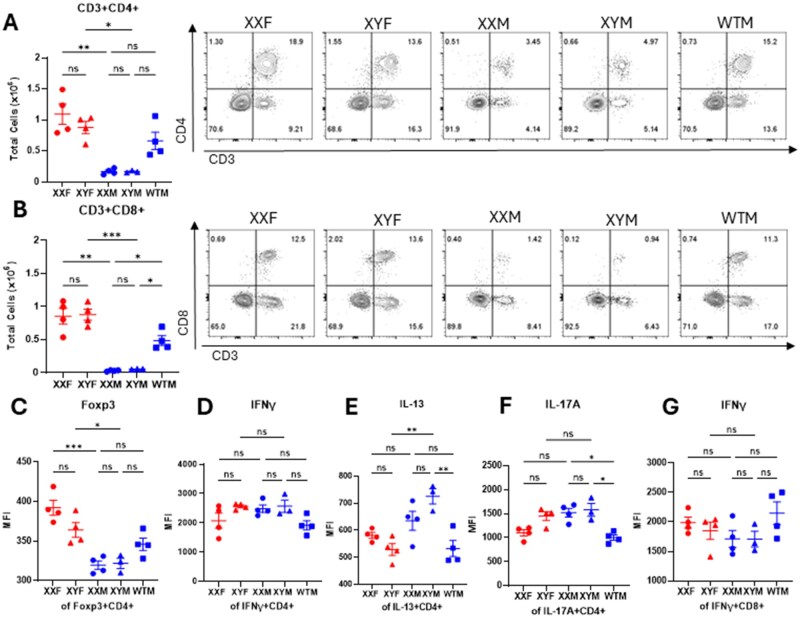
XXM and XYM FCG male mice have a deficit in their CD3^+^CD4^+^ and CD3^+^CD8^+^ Cells Following HDM-Induced Airway Inflammation. (A) Quantification and representative flow diagrams of CD3^+^CD4^+^ cells after HDM challenge. (B) Quantification and representative flow diagrams of CD3^+^CD8^+^ cells after HDM challenge. (C–F) MFI expression of Foxp3, IFN-γ, IL-13, and IL-17A in lung CD3^+^CD4^+^ cells. (G) MFI expression of IFN-γ in lung CD3^+^CD8^+^ cells after HDM challenge. Data are expressed as mean ± SEM: *n* = 3–5 mice per group. Two-way ANOVA with Tukey’s post hoc. * *P* < 0.05, ** *P* < 0.01, *** *P* < 0.001.

While known sex differences exist in allergy-induced airway inflammation in adult mice, with male mice having decreased total lung Th2 and Th17 cells following allergen challenge compared to females,^24–26^^,^^32^^,^^37^ the difference in T cell numbers is not to the extent we observed in the FCG mice. Thus, we sought to compare lung CD4^+^ and CD8^+^ T cell numbers and phenotypes in FCG male mice to WT male mice utilizing a house dust mite (HDM)-induced allergic airway inflammation model, which is well established in our lab, with BALs, lungs, and serum harvested 24 h after the last HDM challenge (Fig. 3A). XXF had significantly higher lung IL-13 and IL-17A compared to XXM mice following HDM sensitization and challenge (Fig. 3B, C). Additionally, HDM-induced lung IFN-γ levels were low and below the limit of detection for all groups of mice (Fig. 3D). Similar to RSV infections in adult mice, HDM-induced infiltration of neutrophils was increased in XXF mice compared to XXM mice (Fig. 3E), but there were no differences in total BAL cells, macrophages, eosinophils, and lymphocytes between the HDM challenged group groups (Fig. 3F, G, and Fig. S6A). We then determined CD4^+^ T cell and CD8^+^ T cell numbers in the lungs following HDM challenge by flow cytometry using the flow gating strategy shown in Fig. S2 and found a similar trend, where XXF and XYF mice had increased CD3^+^CD4^+^ and CD3^+^CD8^+^ T cells compared to XXM and XYM FCG mice (Fig. 4A, B). Remarkably, while not increased to the level of T cells in the XXF and XYF mice, the WTM mice had increased CD4^+^ and CD8^+^ T cells compared to the XXM and XYM FCG mice, demonstrating an exaggerated male phenotype in the FCG male mice. There was a similar pattern in total Tregs, Th1 cells, Th2 cells, and IFN-γ^+^CD8^+^ T cells, whereas WTM mice had similar Th17 cell numbers to FCG male mice (Fig. S6B, C). There were no significant differences between TCR-γδ T cells, NK cells, or IFN-γ^+^ NK cells between the groups (Fig. S6C). As with the RSV infection, there was no significant difference in IFN-γ expression in CD4^+^ and CD8^+^ T cells between groups. Foxp3 expression was increased in Tregs in XXF and XYF and WTM compared to FCG males, while IL-13 and IL-17A were increased in Th2 cells and Th17 cells, respectively, in FCG males compared to WT males (Fig. 4C), indicating that while the number of T helper cells is decreased in FCG male mice, there is no deficit in cytokine production in the context of allergic airway inflammation. In summary, these data demonstrate a deficiency in lung CD4^+^ and CD8^+^ T cells in response to allergen-induced inflammation in XXM and XYM FCG mice compared to FCG females and WTM mice.

### Scarcity of CD4^+^ and CD8^+^ T cells exists in peripheral organs at baseline in XXM and XYM FCG mice compared to WT males and females

**Figure 5:**
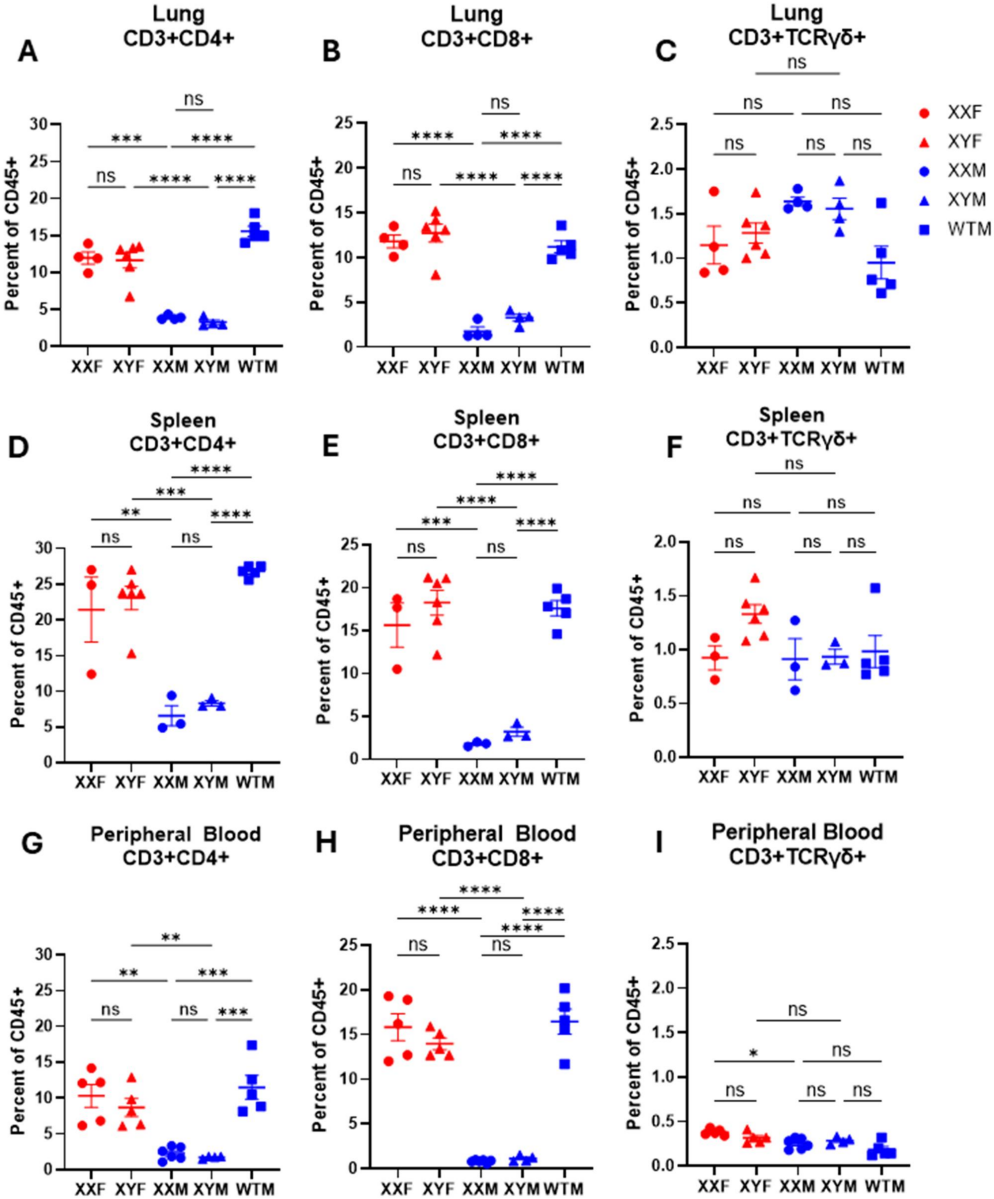
XXM and XYM FCG male mice have a deficit in their CD3^+^CD4^+^ and CD3^+^CD8^+^ cells at baseline in the periphery. Lungs, spleens, and peripheral blood were harvested from adult, FCG mice and WT male mice at baseline. (A–C) Percent of lung CD4^+^, CD8^+^, and TCR-γδ T cells (pre-gated on viable CD45^+^ cells). (D–F) Percent of splenic CD4^+^, CD8^+^, and TCR-γδ T cells (pre-gated on viable CD45^+^ cells). (G–I) Percent of peripheral blood CD4^+^, CD8^+^, and TCR-γδ T cells (pre-gated on viable, CD45^+^ cells). Data are expressed as mean ± SEM: *n* = 3–6 mice per group from independent experiments. Spleen and lungs were obtained from the same cohort of mice and peripheral blood a separate cohort. Two-way ANOVA with Tukey’s post hoc. * *P* < 0.05, ** *P* < 0.01, *** *P* < 0.001, **** *P* < 0.0001, NS, not significant.

Because we found lower CD4^+^ and CD8^+^ T cells in the FCG male mice compared to WT male mice in the lungs following allergic airway inflammation, we sought to determine if these differences would remain at baseline in the absence of infection or immune challenge in the lungs, spleen, and peripheral blood. Tissues and blood were harvested from adult XXF, XYF, XXM, XYM, and WTM mice and analyzed for flow cytometry to identify CD4 T cell subsets and other immune cells (gating strategies shown in Figs. S7–9). We observed a similar pattern, with XXM and XYM having decreased percents of lung CD4^+^ and CD8^+^ T cells compared to female FCG mice and WTM mice (Fig. 5A, B), while there were no deficits in TCR-γδ T cells (Fig. 5C). FCG male mice did not have a deficiency in other immune cell subsets in the lungs, including B cells, innate lymphoid cells (ILCs), NK cells, and myeloid cells, and their percent of other CD45^+^ cells were higher in some instances, likely an inflated number due to their lack of T cells (Fig. S10A–D). We then conducted flow cytometry on harvested spleens, a peripheral immune organ, and found a deficit in CD4+ and CD8^+^ T cells in the XXM and XYM FCG male mice, similarly to what has been previously reported,^52^ but no significant differences in TCR-γδ T cells (Fig. 5D–F), similar to the lungs. There was also no deficiency in other immune cell subsets in the spleen (Fig. S10E–H). This deficit in T cells could be due to their inability to migrate to and enter peripheral immune organs, or it could be due to something further upstream. To answer this question, we isolated peripheral blood from FCG mice and WT male mice and found that the CD4^+^ T cell and CD8^+^ T cell deficiency persists in the peripheral blood (Fig. 5G–I) while there is not insufficiency in other immune cell subsets (Fig. S10I–L). This suggests that the decrease in CD4^+^ and CD8^+^ T cells in the XXM and XYM FCG mice is not due to the inability of T cells to enter peripheral organs.

### Thymic T cell development and bone marrow progenitors are similar in FCG mice

**Figure 6:**
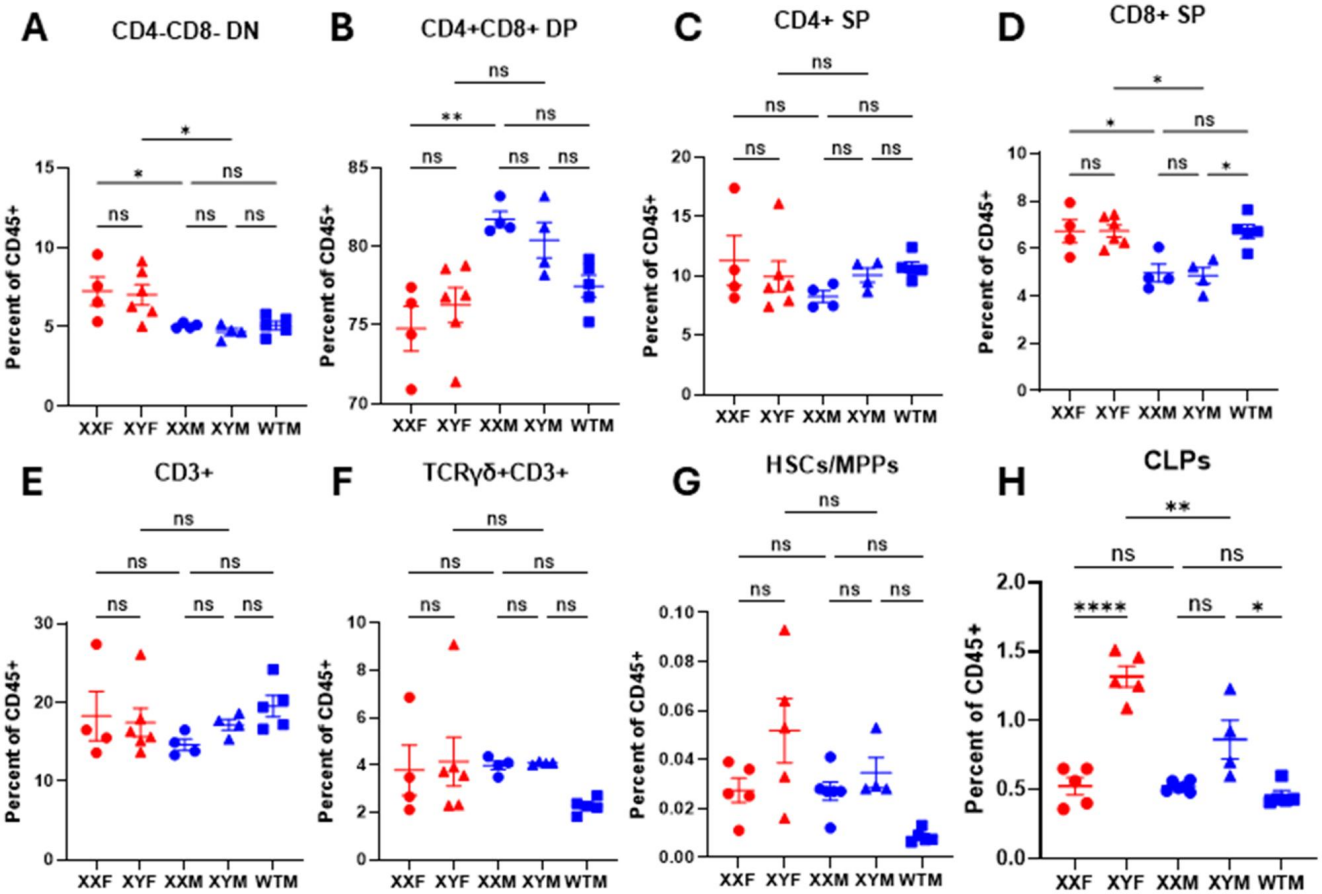
Thymic T cell development and bone marrow progenitors are similar in FCG mice. (A–F) Thymuses were harvested from adult, FCG mice and WT male mice at baseline and the percentages of T cells were determined as a percentage CD45^+^ cells. (A) CD4^−^CD8^−^ double negative (DN) T cells, (B) CD4^+^CD8^+^ double positive (DP) T cells, (C) CD4^+^ single positive (SP) T cells, (D) CD8^+^ SP T cells, (E) overall CD3^+^ T cells, and (F) TCR-γδ^+^ T cells. (G–H) Bone marrow was also harvested from adult, FCG mice and WT male mice at baseline and hematopoietic stem cells (HSC) and/or multipotent progenitor cells (MPP) and common lymphoid progenitors (CLPs) were determined. Data are expressed as mean ± SEM: *n* = 4–6 mice per group. Two-way ANOVA with Tukey’s post hoc, * *P* < 0.05, ** *P* < 0.01, **** *P* < 0.0001, NS, not significant.

Given differences in T cell abundance in the peripheral blood, we next wanted to determine if the FCG mice had differences in T cells in the thymus or in bone marrow progenitor cells (gating strategies shown in Figs. S11–12). Similar to prior results,^52^ we found no significant differences in CD3^+^ T cell abundance in the thymus among the FCG groups (Fig. 6E). There were also no differences in CD4^+^ single positive (SP) cells; however, there was a trend toward lower CD4^−^CD8^−^ double negative (DN) cells and an increase in CD4^+^CD8^+^ double positive (DP) cells in the XXM, XYM, and WTM, whereas there was a significant decrease in CD8^+^ single positive (SP) cells in the XXM and XYM compared to females and WTM (Fig. 6A–D). The increase in CD4^+^CD8^+^ DP cells and decrease in CD8^+^ SP cells in the FCG male mice could indicate a slight defect in later stages of T cell maturation in the thymus, potentially in positive selection.^53^ TCR-γδ^+^ CD3^+^ T cells were also not different between the groups (Fig. 6F). We also examined bone marrow progenitor cells and determined that Lin-Sca-1^+^c-Kit^+^ bone marrow progenitors (hematopoietic stem cells [HSCs] and multipotent progenitors [MPPs]) were not significantly different between the groups (Fig. 6G). Interestingly, common lymphoid progenitors (CLP) were increased in XYF and XYM FCG mice but not WTM mice, suggesting that an alteration in the Y chromosome in the FCG mice may be driving a difference in CLP frequency (Fig. 6H). A recent study reported a translocation of a copy of the X chromosome to the Y(*Sry-*) chromosome in the FCG mice,^54^ which could potentially explain this separate phenomenon. Overall, these results suggest that deficiencies in peripheral T cells in the FCG male mice are unlikely due to differences in T cell development in the thymus or in bone marrow progenitor cells.

## Discussion

Sex differences in immune response to infections, autoimmune diseases, and allergy and asthma are well documented. The FCG mouse model system provides the opportunity to separate the effects of gonadal hormones and sex chromosomes in disease.^46^ Previous studies using the FCG model demonstrated differential contributions of gonadal hormones versus sex chromosomes in infection,^55^ autoimmune disease,^56^^,^^57^ and delayed hypersensitivity reactions and allergic responses.^58^^,^^59^

A deficiency in T cells in the periphery in XXM and XYM compared to XXF and XYF FCG mice has been described previously by other groups.^52^^,^^58^ XXM and XYM FCG mice had a deficiency in CD4^+^ and CD8^+^ T cells in the popliteal lymph nodes that were eliminated by gonadectomy at 3 wk of age.^58^ Additionally, neonatal and adult gonadectomized XXM and XYM mice had decreased CD4^+^ and CD8^+^ T cells, but not B cells, in the spleens and peripheral blood,^52^ with no difference in total SP CD4^+^ and CD8^+^ T cells in the thymus of young and gonadectomized adult FCG male mice or in immune progenitors in the neonatal spleen.^52^ Our current work corroborated these findings and expanded on them by including a WT male mouse in most experiments as a comparator group for the FCG male mice, characterizing T cell populations in the lung, a non-lymphoid organ, both at baseline and in the context of viral infection or allergen challenge, and demonstrating a dampened immune response in the XXM and XYM FCG mice. Our study also builds upon these data by including additional immune cell subsets in our analyses, showing no major differences in TCR-γδ T cells, NK cells, ILCs, and myeloid cells.

While we have extensively characterized the CD4^+^ and CD8^+^ T cell deficit in the peripheral blood and organs of FCG XXM and XYM mice at baseline and in models of immune activation, our study did not determine why FCG XXM and XYM have reduced CD4^+^ and CD8^+^ T cells in the periphery. We did determine that TCR-γδ^+^ T cell abundance in FCG and WTM males is similar. TCR-γδ^+^ T cells are fully developed at the third double negative (DN3) stage in the thymus,^53^ making it likely that defects in T cell development occur downstream of the DN3 stage. We determined an increase in the percentage of CD4^+^CD8^+^ DP T cells and a trend for a decrease in CD8^+^ SP T cells in the thymus of FCG male mice. DP T cells undergo positive selection for interaction with self MHC before becoming CD4^+^ or CD8^+^ SP T cells.^53^ Thus, an impairment in positive selection could also explain the defect in peripheral T cells in the FCG males. Interestingly, there was no sex difference in DP or SP T cells in the thymus of FCG pups at baseline,^52^ suggesting this difference may develop in mature FCG mice.

Egress from the thymus involves sphingosine-1-phosphate (S1P) binding to sphingosine-1-phosphate receptor 1 (S1P1R) on T cells,^60^^,^^61^ and *S1pr1* is encoded on chromosome 3 in mice, and we wondered whether this was disrupted by the overexpression of *Sry* on chromosome 3. However, total *S1pr1* expression was similar in thymuses from FCG and WTM mice (data not shown), and thymic weights were similar between groups (data not shown). This would suggest that egress from the thymus is not impaired in the XXM and XYM FCG mice, but whole thymic expression may not be specific enough to detect cell-specific differences. Another group showed using flow cytometry that female FCG mice had significantly elevated numbers of S1p1r and S1p3r expressing CD4^+^ T cells and a greater expression of S1p1r on a per cell basis on CD8^+^ T cells by MFI in the thymus compared to FCG males,^52^ suggesting that *Sry* overexpression may be affecting S1p1r transcription, translation, or post-translational trafficking to the cell surface in T cells specifically. Thus, in future studies it would be interesting to determine if this difference exists between female and wildtype males. Other possible explanations for the defect of CD4^+^ and CD8^+^ T cells in the periphery could be a deficit in T cell survival signaling or T cell proliferation or an increase in apoptotic signals leading to lower T cell numbers in the peripheral blood and organs. Given all these possibilities, more work will be needed to understand the full mechanism underlying the deficit of T cells in the FCG male mice.

Although circulating testosterone levels between adult FCG males and WT males are similar^51^^,^^62^ and gonadectomy in adult FCG did not eliminate this T cell deficit,^52^ another possible explanation for the T cell deficit is differences in the perinatal testosterone surge between XXM and XYM FCG mice and WTM mice. A perinatal testosterone surge occurs in phenotypic male mice (XXM, XYM, and WTM) and imparts permanent systemic effects,^63^^,^^64^ and studies have shown a deficit in CD4^+^ and CD8^+^ T cells in the periphery in neonatal mice.^52^^,^^65^ However, other studies have found that castration at 3 wk of age reverses the exaggerated male phenotype in the FCG XXM and XYM mice in lymph nodes,^58^ indicating that the reversible effects of other testicular secretions, which could be affected by *Sry* expression or overexpression,^66^^,^^67^ may be affecting T cell number, rather than an early testosterone surge, or that reversible effects of testicular secretions could be tissue specific and affect immune cell trafficking through the lymph nodes but not the spleen.^52^ Testicular secretions beyond testosterone include inhibin and activin, which have been implicated in affecting T cell function.^68–70^ Finally, several immune-related genes are expressed on mouse chromosome 3, including *S1pr1*, *Il2*, *Il7*, *Lag3*, and *Lef,*^71^ and the insertion of 12–14 copies of the *Sry* gene on chromosome 3^46^^,^^51^ could be disrupting immune gene enhancer or promoter regions, therefore affecting their expression. Future studies on how *Sry* translocation affects T cell numbers in the spleen, blood, and peripheral tissues are warranted.

The FCG mouse model remains a useful model for separating the effects of sex chromosomes and sex hormones on disease physiology and pathology in many disease contexts. Furthermore, the exaggerated male phenotype due to *Sry* overexpression in the FCG model could be beneficial for deeper study of basic immune mechanisms underlying sex differences in disease and regulation of immune cell distribution. However, our study demonstrates that caution should be taken in not generalizing these findings to normal male physiology especially in the context of T cell–mediated diseases.

## Supplementary Material

vkag207_Supplementary_Data

## Data Availability

All data are present within the manuscript and included within the figures and supplemental files. Any additional information is available from the lead contact upon reasonable request.
