# Supplementary material for: Deficiency in circulating T cells in four core genotypes mice with Sry translocation
Source: J Immunol. 2026 Aug 13;215(8):vkag207. doi: 10.1093/jimmun/vkag207 (PMC13472664; doi:10.1093/jimmun/vkag207)
Supplement: Supplementary file 1 [file vkag207_supplementary_data.zip › JI SUPPLMENTAL FIGURES REVISED.pdf]

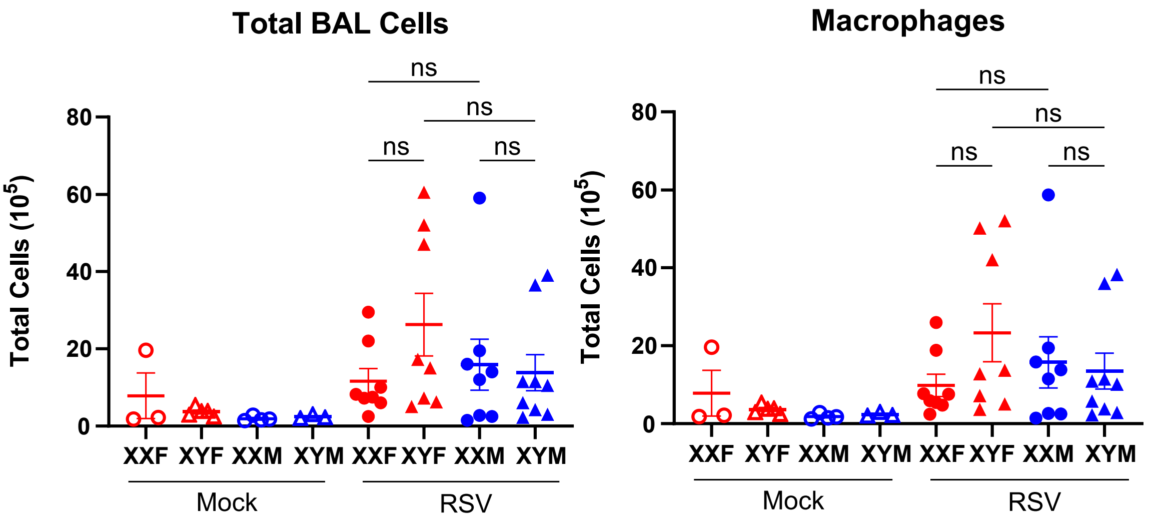

Supplemental Figure 1. Infiltration of BAL Inflammatory Cells in FCG Mice Following RSV Infection (Related to Figure 1). Quantification of total BAL cells and BAL macrophages was determined 7 dpi. Data are expressed as mean ± SEM: n= 6- 9 mice per group combined from 2 independent experiments. Two-way ANOVA with Tukey's post-hoc, ns: not significant.

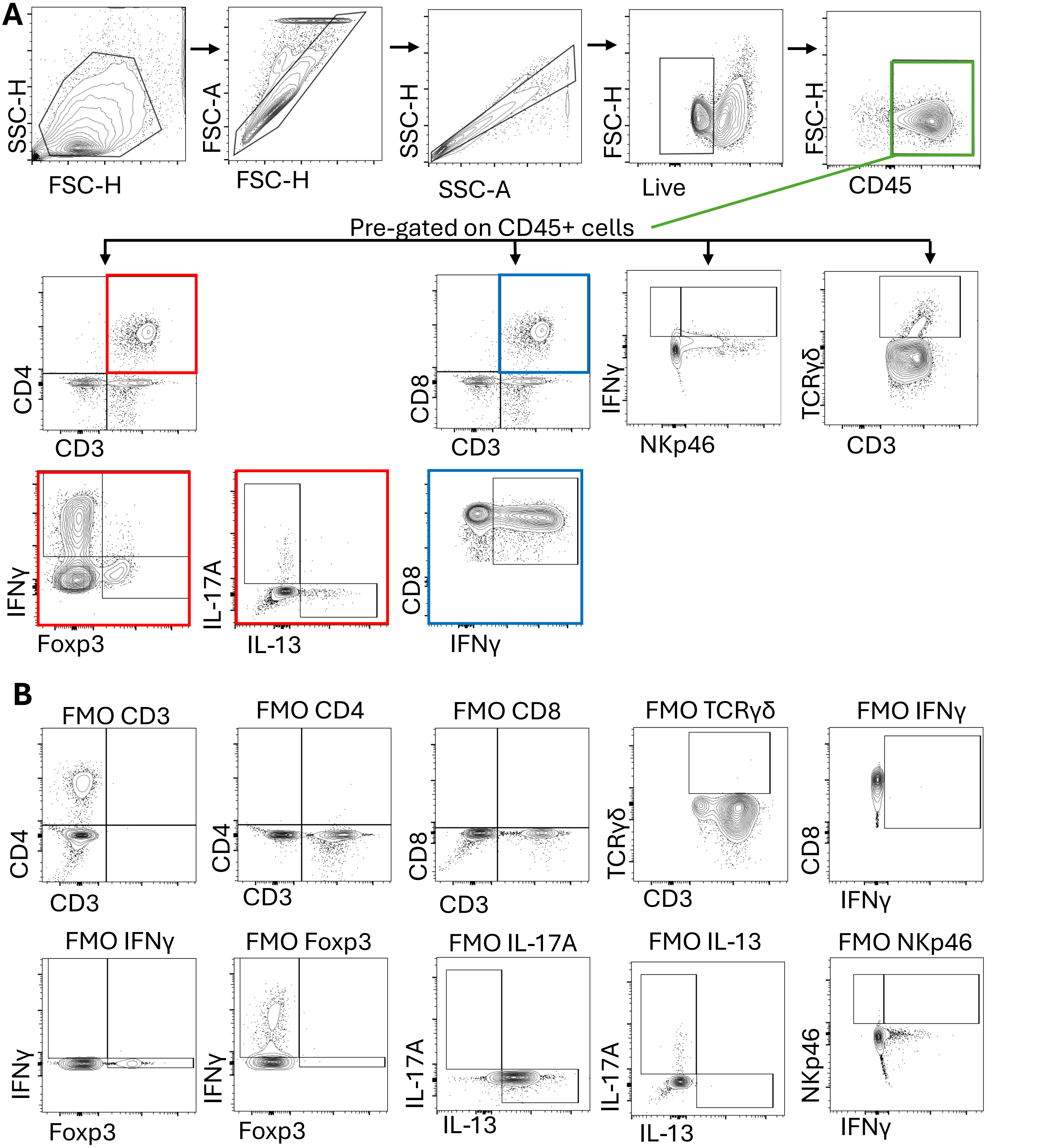

Supplemental Figure 2. Gating strategy for identification of Th1, Th2, Th17, Tregs, CD8 T cells, and NK cells in RSV-infected or HDM-challenged mice (Related to Figures 1-4 and Supplemental Figures 3-6). (**A**) Gating strategy for lung CD3+CD4+ T cells and subsets (Th1, Th2, Th17, and Tregs), CD3+CD8+ T cells, IFNγ+CD8+ T cells, NK cells (CD3-NKp46+) and IFNγ+ NK Cells from a representative XXF sample. (**B**) FMOs for gating strategy.

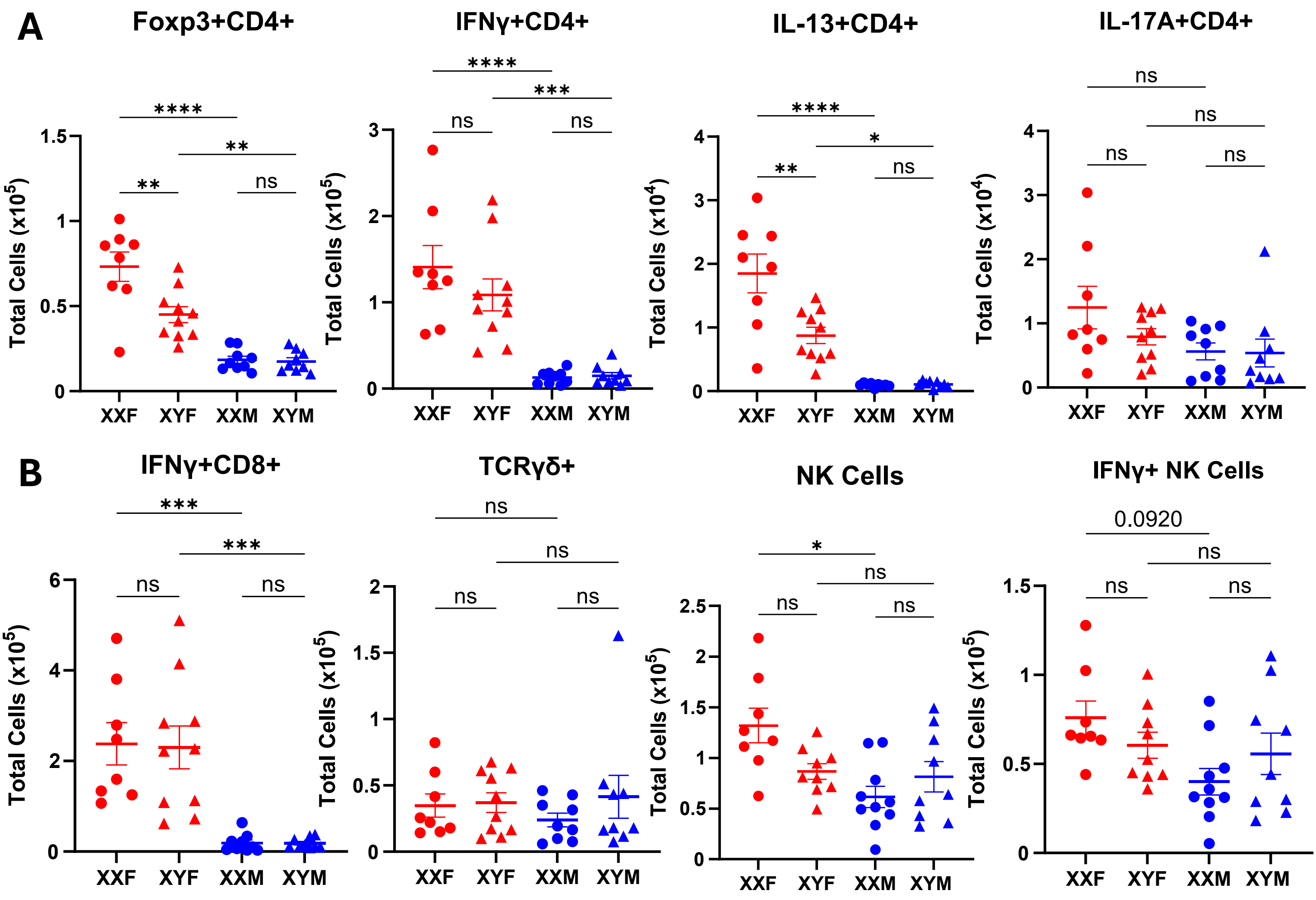

Supplemental Figure 3. XXM and XYM FCG Male Mice have a Deficit in their CD3+CD4+ and CD3+CD8+ Cells in the Lungs Following RSV Infection (Related to Figure 2). (A) Quantification of lung CD3+CD4+ T helper cell subsets 7 dpi: Tregs (Foxp3+CD4+), Th1 (IFNγ+CD4+), Th2 (IL-13+CD4+), and Th17 (IL-17A+CD4+). (**B**) Quantification of IFNγ+CD8+, TCRγδ, total NK Cells, and IFNγ+ NK cells 7dpi. Data are expressed as mean ± SEM: n= 6- 9 mice per group combined from 2 independent experiments. Two-way ANOVA with Tukey's post-hoc, * $p < 0.05$, ** $p < 0.01$, *** $p < 0.001$, **** $p < 0.0001$, ns: not significant.

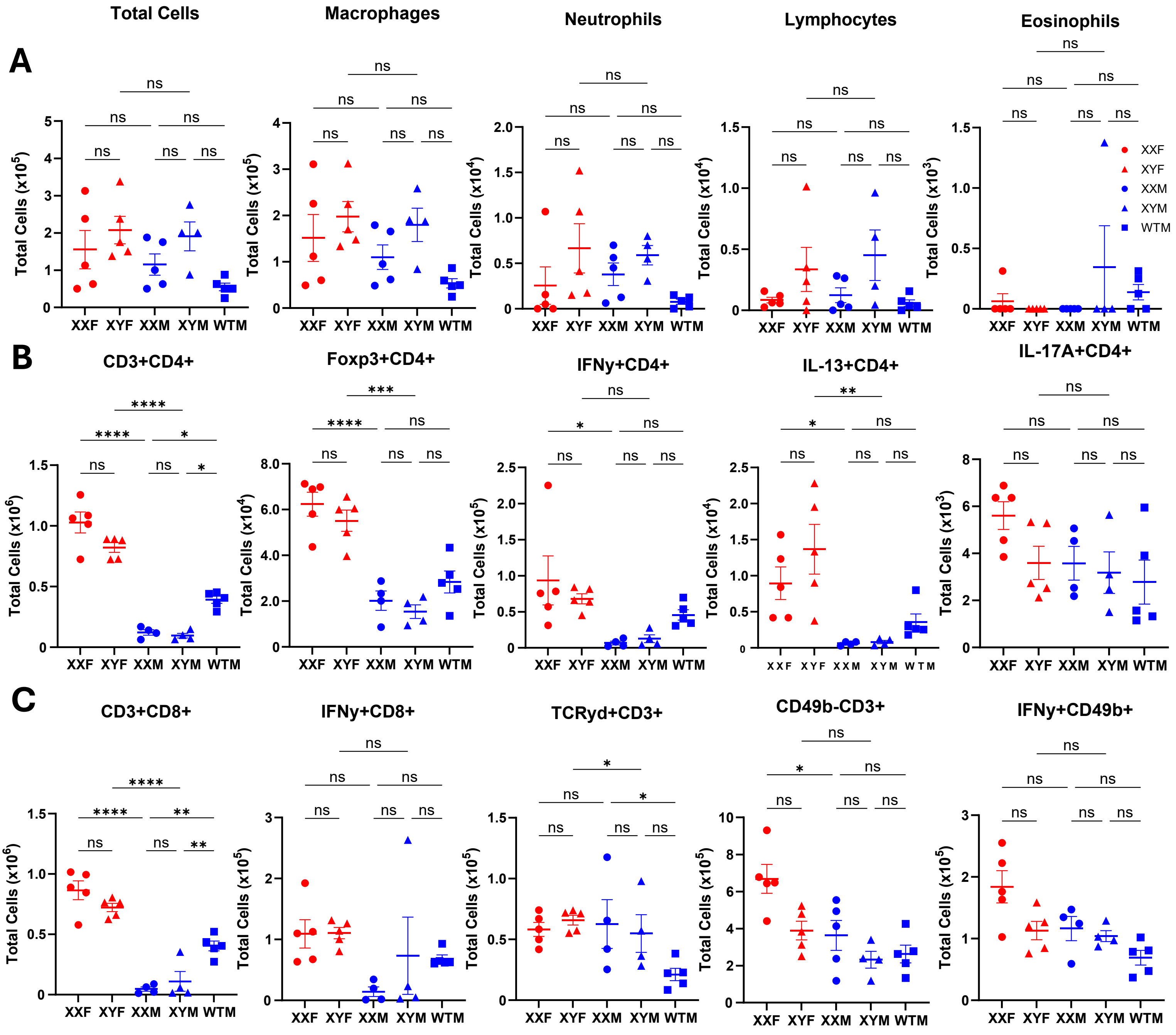

Supplemental Figure 4. XXM and XYM FCG Male Mice have a Deficit in their CD3+CD4+ and CD3+CD8+ Cells in the Lungs and No Difference in Overall Lung Inflammation 4 Days Post Infection with RSV. (A) Quantification of total BAL cells, macrophages, neutrophils, lymphocytes, and eosinophils in the BAL fluid 4 dpi. (**D**) Quantification of lung CD3+CD4+ T cells and CD3+CD4+ T helper cell subsets: Tregs (Foxp3+CD4+), Th1 (IFNγ+CD4+), Th2 (IL-13+CD4+), and Th17 (IL-17A+CD4+). (**C**) Quantification of total lung CD3+CD8+ T cells, IFNγ+CD8+ T cells, TCRγδ, total NK Cells, and IFNγ+ NK cells. Data are expressed as mean ± SEM: n= 4- 5 mice. Two-way ANOVA with Tukey's post-hoc, * $p < 0.05$, ** $p < 0.01$, *** $p < 0.001$, **** $p < 0.0001$ ns: not significant.

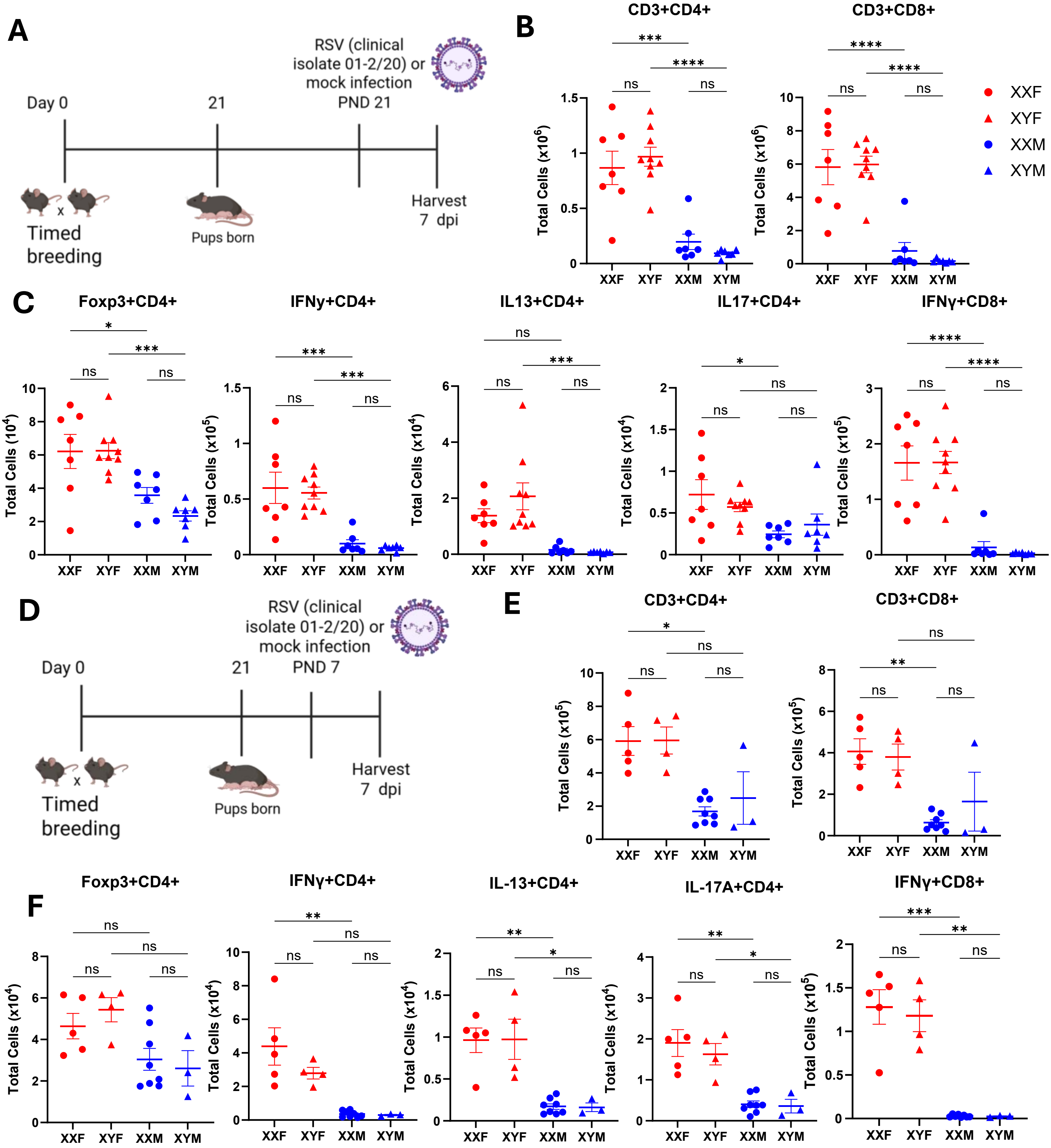

Supplemental Figure 5. Young Pre-pubertal XXM and XYM FCG Male Mice have a Deficit in their CD3+CD4+ and CD3+CD8+ Cells Following RSV Infection in the Lungs. (A) Model for PND 21 of RSV infection. Timed breedings were set up and XXF, XYF, XXM, and XYM 21-day-old pups were infected with RSV or mock and lungs harvested 7 dpi. (**B**) Total lung CD3+CD4+ and CD3+CD8+ T cells in the lungs 7 dpi. (**C**) Treg, Th1, Th2, Th17, and IFNγ+CD8+ effector T cell counts in the lungs 7 dpi. (**D**) Model for PND 7 of RSV infection. Timed breedings were set up and XXF, XYF, XXM, and XYM 7-day-old pups were infected with RSV or mock and lungs harvested 7 dpi. (**E**) Total lung CD3+CD4+ and CD3+CD8+ T cells in the lungs 7 dpi at 14 days old. (**F**) Treg, Th1, Th2, Th17, and IFNγ+CD8+ effector T cell counts in the lungs 7 dpi at 14 days old. Data are expressed as mean ± SEM: n= 7- 9 mice per group combined from 2 independent experiments (A-C) and n= 3-8 from 1 independent experiment. Two-way ANOVA with Tukey's post-hoc, * $p < 0.05$, ** $p < 0.01$, *** $p < 0.001$, **** $p < 0.0001$, ns: not significant.

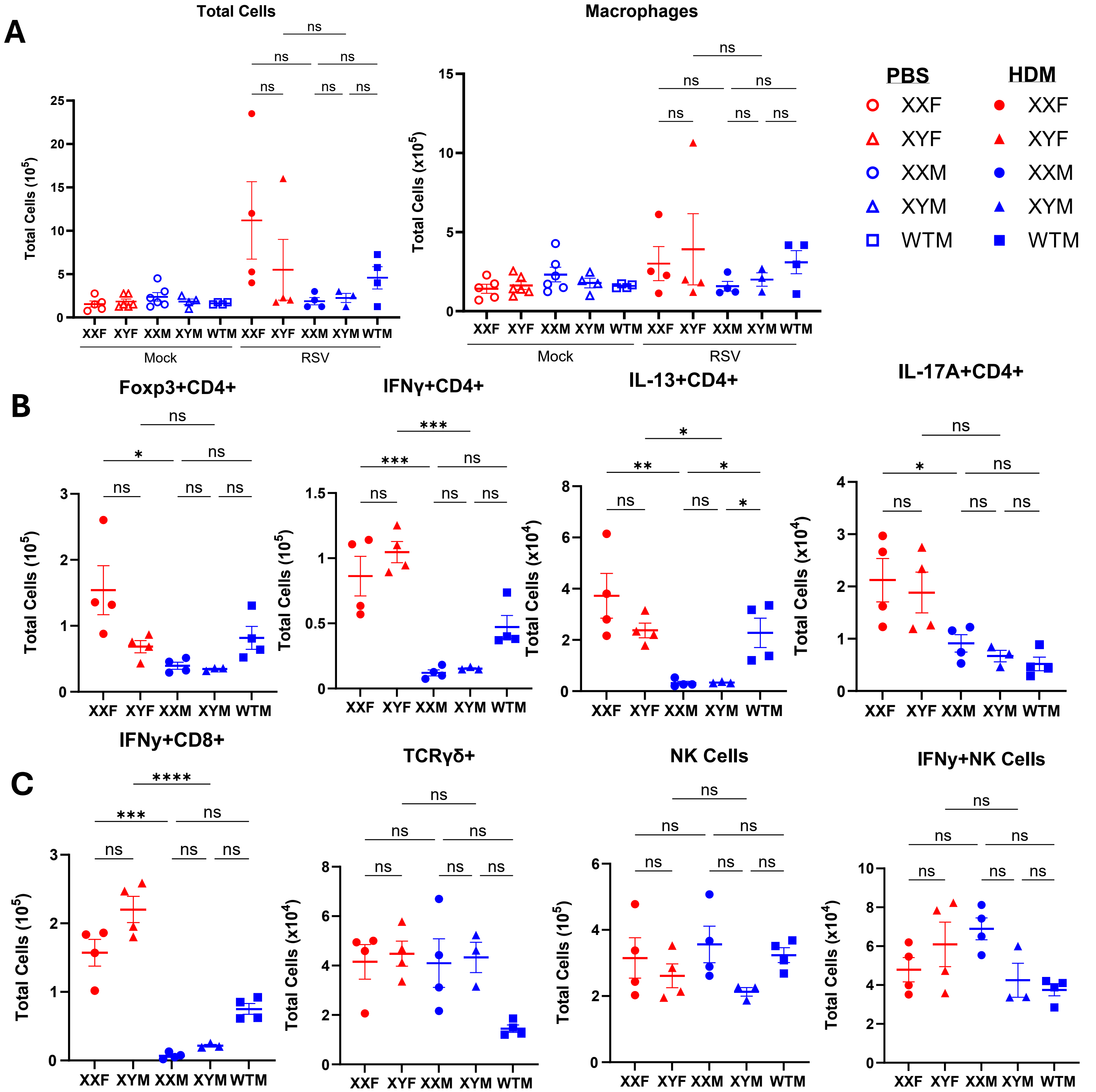

Supplemental Figure 6. XXM and XYM FCG Male Mice have a Deficit in their CD3+CD4+ and CD3+CD8+ Cells Following HDM-Induced Airway Inflammation (Related to Figure 3- 4). (A) Quantification of total BAL cells and BAL macrophages. (**B**) Quantification of lung CD3+CD4+ T helper cell subsets: Tregs (Foxp3+CD4+), Th1 (IFNγ+CD4+), Th2 (IL-13+CD4+), and Th17 (IL-17A+CD4+). (**C**) IFNγ+CD8+, TCRγδ, total NK Cells, and IFNγ+ NK cells. Data are expressed as mean ± SEM: n= 3- 5 mice. Two-way ANOVA with Tukey's post-hoc, * $p < 0.05$, ** $p < 0.01$, *** $p < 0.001$, **** $p < 0.0001$ ns: not significant.

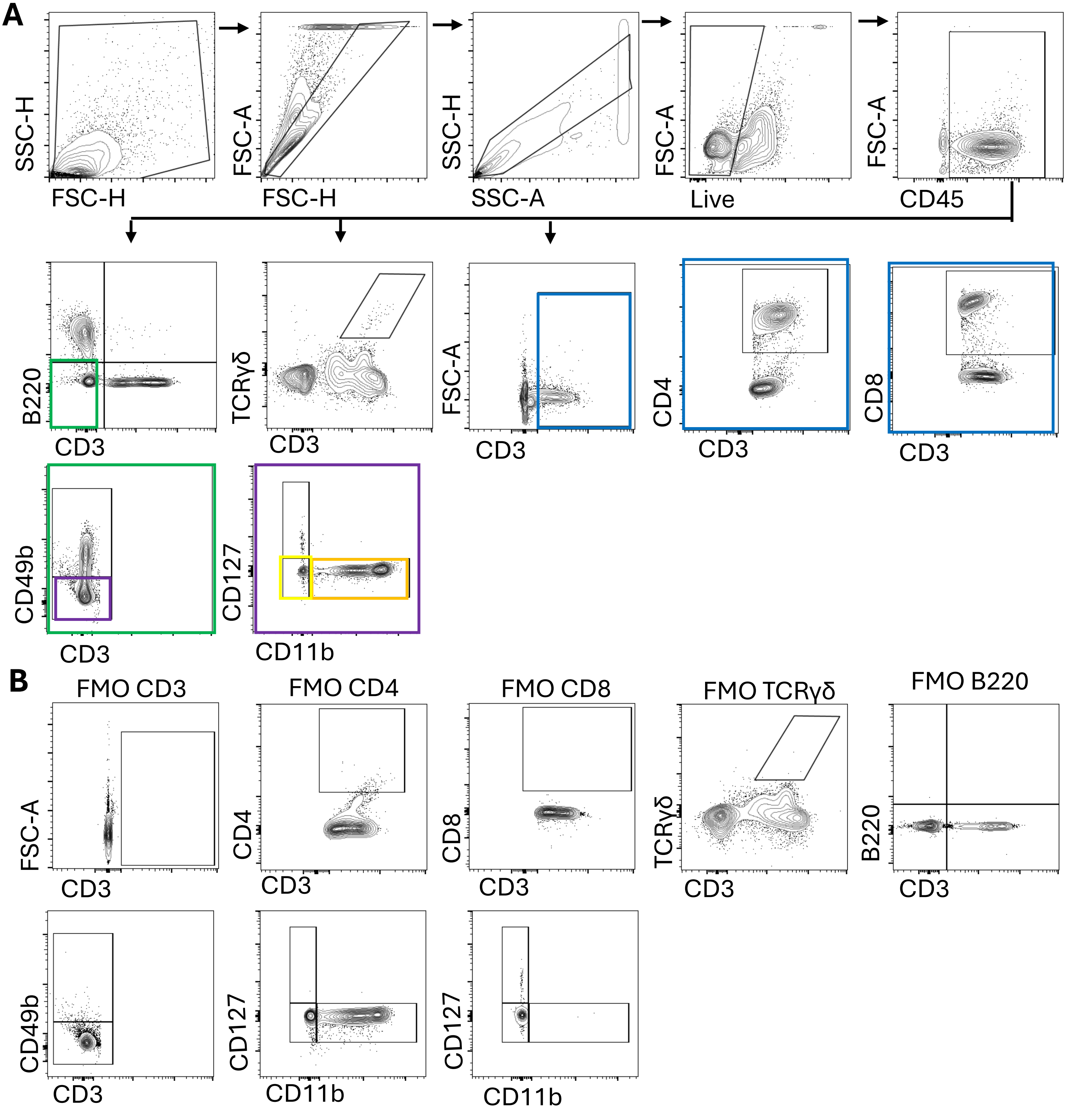

Supplemental Figure 7. Lung gating strategy for identification of CD4+ T cells, CD8+ T cells, TCRγδ+ T cells, B cells, NK Cells, ILCs, and Myeloid Cells (Related to Figure 5). (**A**) Gating strategy for lung CD3+CD4+ T cells, CD3+CD8+ T cells, TCRγδ+ T cells, NK cells (CD3-CD49b+), B cells (CD3-B220+), innate lymphoid cells (ILCs) (CD3-B220-CD49b-CD11b-CD127+), and myeloid cells (CD11b+CD3-CD49b-) from a representative XXF sample. (**B**) FMOs for gating strategy.

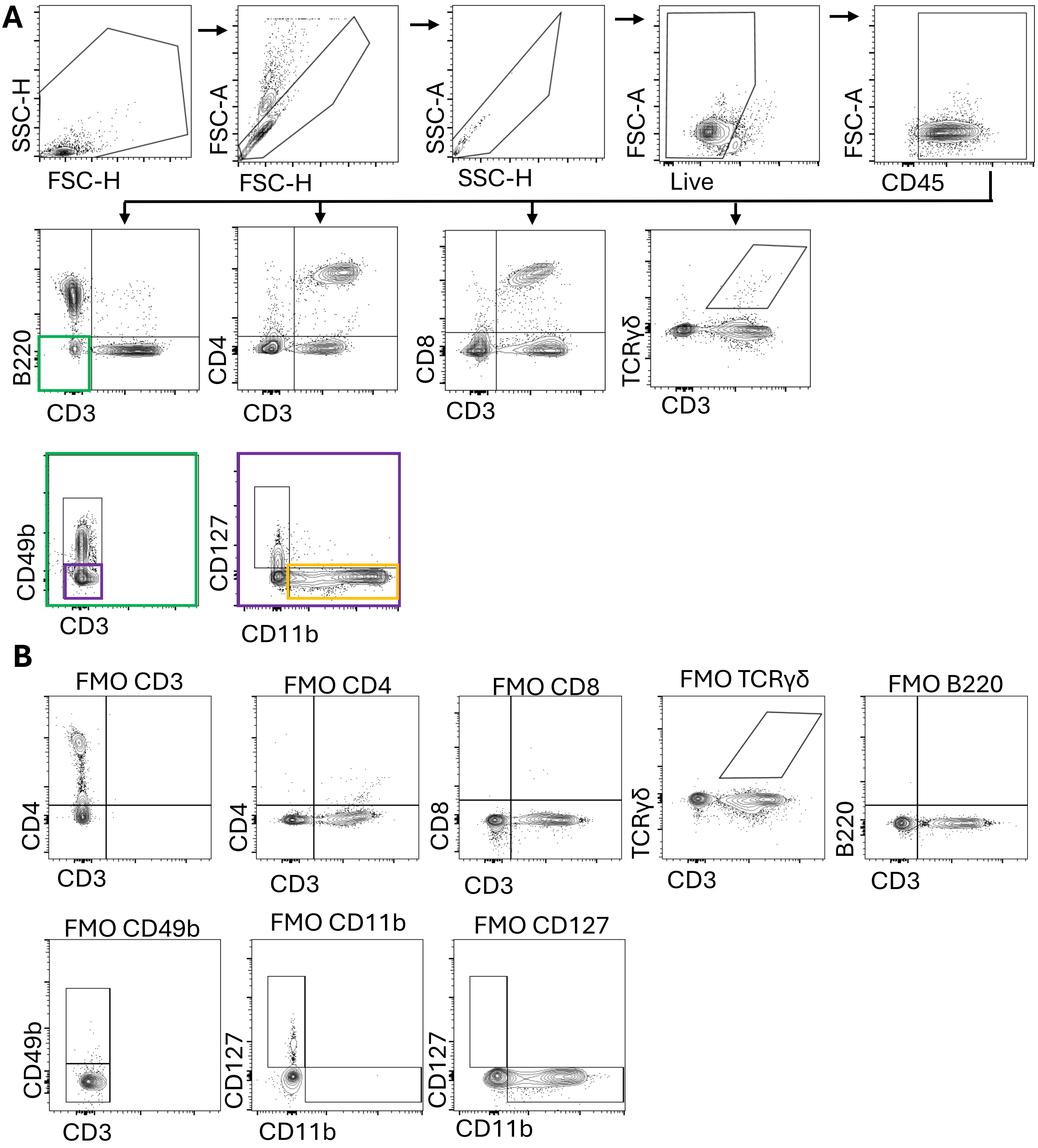

Supplemental Figure 8. Spleen gating strategy for identification of CD4+ T cells, CD8+ T cells, TCRγδ+ T cells, B cells, NK Cells, ILCs, and Myeloid Cells (Related to Figure 5). (**A**) Gating strategy for lung CD3+CD4+ T cells, CD3+CD8+ T cells, TCRγδ+ T cells, NK cells (CD3-CD49b+), B cells (CD3-B220+), innate lymphoid cells (ILCs) (CD3-B220-CD49b-CD11b-CD127+), and myeloid cells (CD11b+CD3-CD49b-) from a representative XXF sample. (**B**) FMOs for gating strategy.

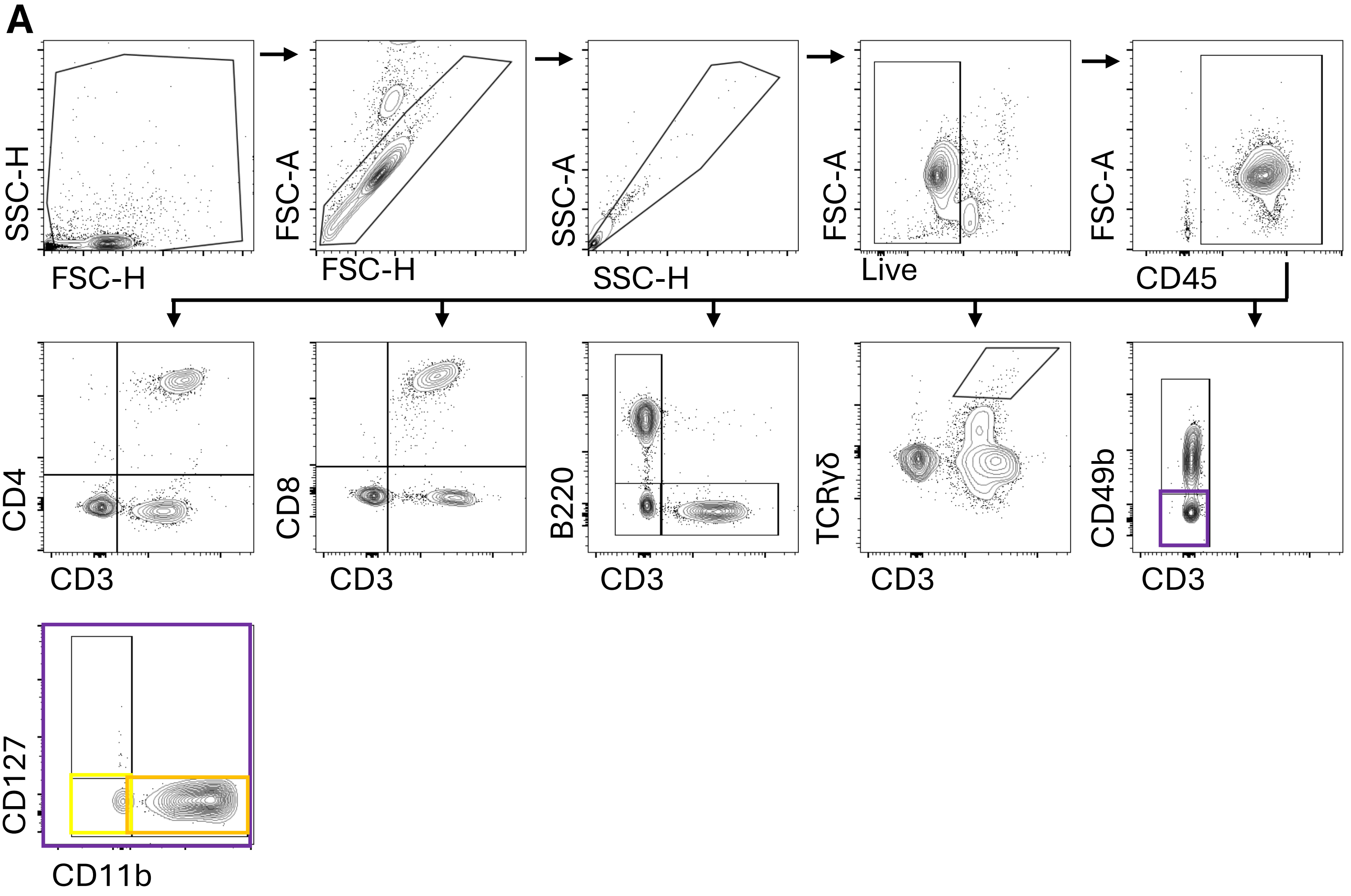

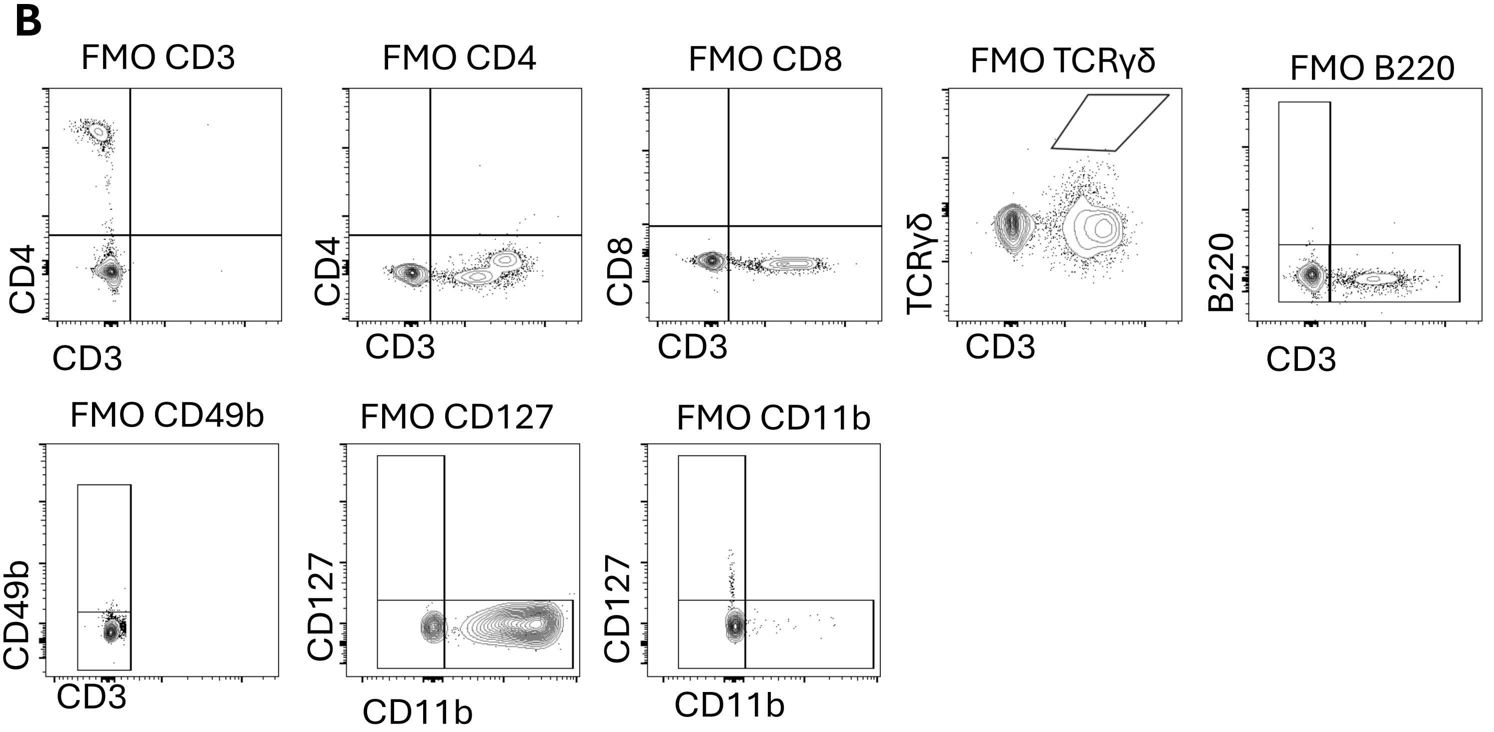

Supplemental Figure 9. Peripheral blood Gating strategy for identification of CD4+ T cells, CD8+ T cells, TCRγδ+ T cells, B cells, NK Cells, ILCs, and Myeloid Cells (Related to Figure 5). (**A**) Gating strategy for lung CD3+CD4+ T cells, CD3+CD8+ T cells, TCRγδ+ T cells, NK cells (CD3-CD49b+), B cells (CD3-B220+), innate lymphoid cells (ILCs) (CD3-B220-CD49b-CD11b-CD127+), and myeloid cells (CD11b+CD3-CD49b-) from a representative XXF sample. (**B**) FMOs for gating strategy.

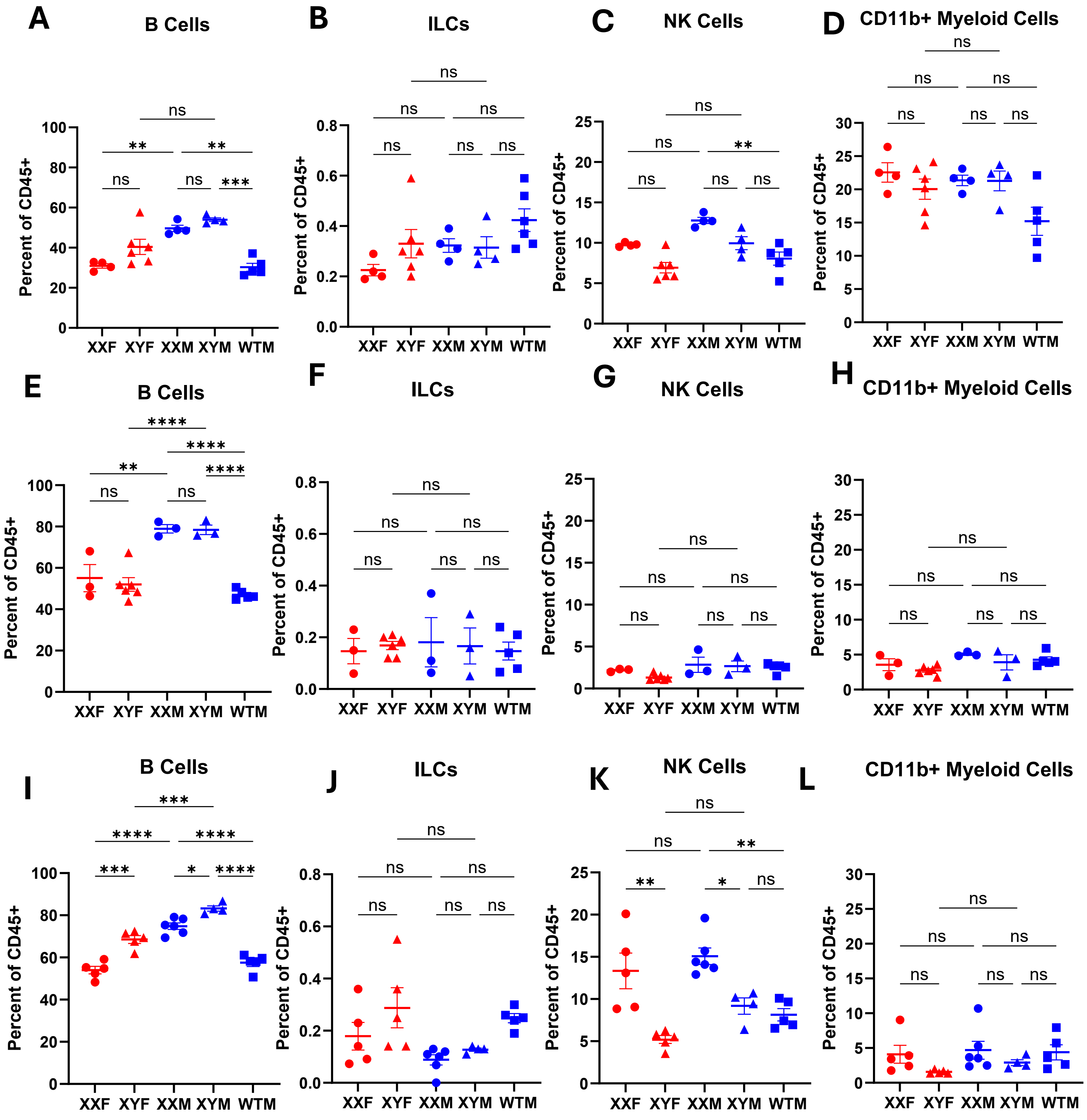

Supplemental Figure 10. Percentages of immune cells in lungs, spleens, and peripheral blood at baseline (Related to Figure 5). Lungs, spleens, and peripheral blood were harvested from adult, FCG mice and WT male mice at baseline. (**A-D**). Percent of lung B cells, ILCs, NK cells, and CD11b+ myeloid cells (pre-gated on viable CD45+ cells). (**E-H**) Percent of splenic B cells, ILCs, NK cells, and CD11b+ myeloid cells (pre-gated on viable CD45+ cells). **(I-L)** Percent of peripheral blood B cells, ILCs, NK cells, and CD11b+ myeloid cells (pre-gated on viable CD45+ cells). Data are expressed as mean ± SEM: n= 3-6 mice per group from independent experiments. Spleen and lungs were obtained from the same cohort of mice and peripheral blood a separate cohort. Data are expressed as mean ± SEM: n= 3- 6 mice per group. Two-way ANOVA with Tukey's post-hoc. * $p < 0.05$, ** $p < 0.01$, *** $p < 0.001$, **** $p < 0.0001$, ns: not significant.

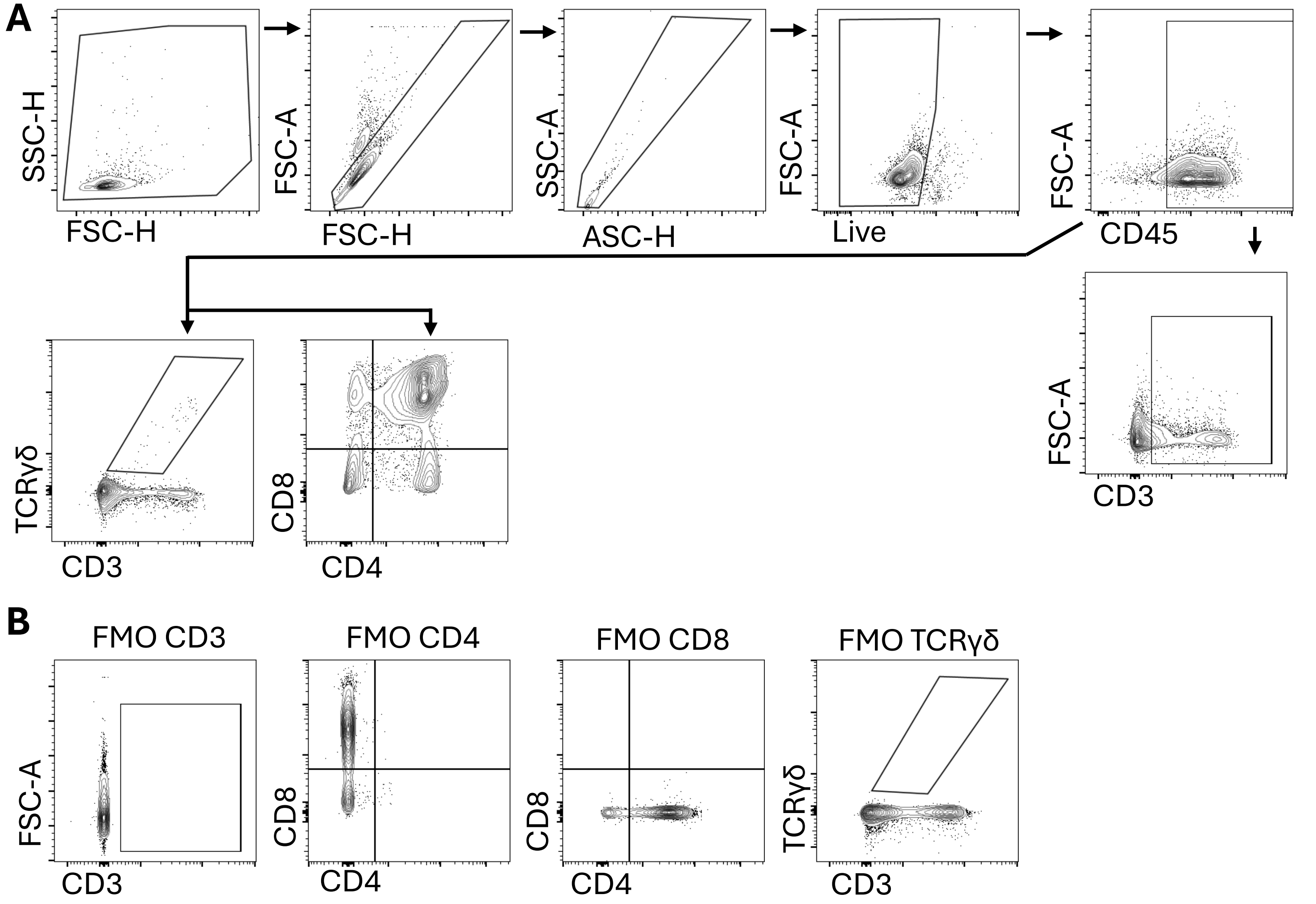

Supplemental Figure 11. Thymus gating strategy for identification of CD4-CD8- DN, CD4+ SP, CD8+ SP, and CD4+CD8+ DP T cells, CD3+ T Cells, TCRγδ+ T cells, NK Cells, Epithelial Cells, and Myeloid Cells (Related to Figure 6). (**A**) Gating strategy for thymus CD3+ T cells, CD4-CD8- DN, CD4+ SP, CD8+ SP, and CD4+CD8+ DN T cells, and TCRγδ+ T cells from a representative XXF sample. (**B**) FMOs for gating strategy.

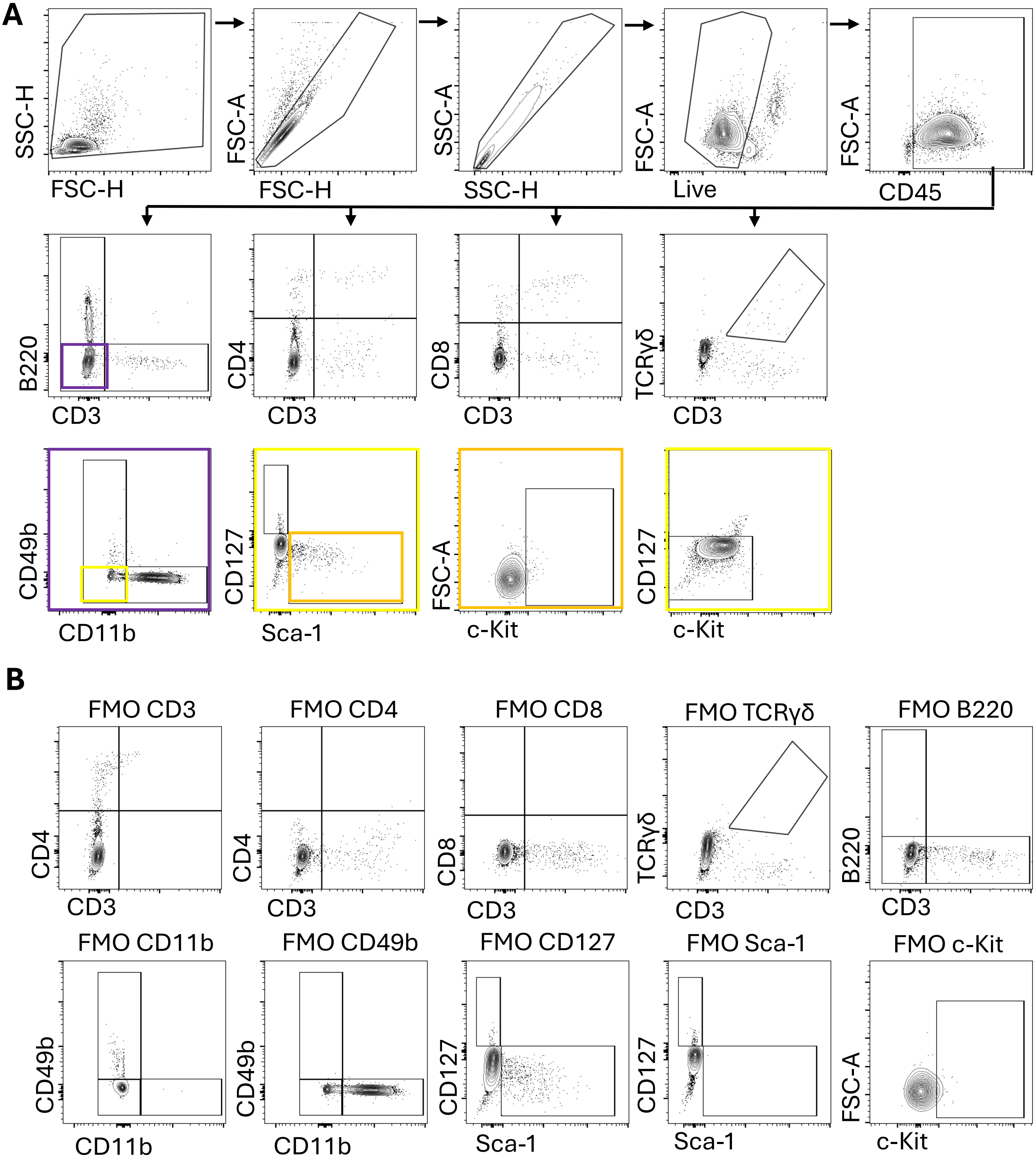

Supplemental Figure 12. Bone marrow gating strategy for identification of CD4+ T cells, CD8+ T cells, TCRγδ+ T cells, B cells, NK Cells, ILCs, Myeloid Cells, and Progenitor cells (Related to Figure 6). (**A**) Gating strategy for lung CD3+CD4+ T cells, CD3+CD8+ T cells, TCRγδ+ T cells, NK cells (CD3-CD49b+), B cells (CD3-B220+), innate lymphoid cells (ILCs) (CD3-B220-CD49b-CD11b-CD127+), myeloid cells (CD11b+CD3-B220-), hematopoietic stem cells and multipotent progenitors (LSK) (Lin-CD127-Sca-1+c-Kit+) and lineage committed common lymphoid progenitors (Lin-CD127-c-Kit-) from a representative XXF sample. (**B**) FMOs for gating strategy.

Supplemental Table 1. Surface antibodies used in this project.

Antibody	Manufacturer	Catalog Number
Anti-Mouse CD3 APC-Cy7	BD Biosciences	557596
Anti-Mouse CD4 PE-Cy5	Thermo Fisher Scientific	15-0041-82
Anti-Mouse CD45 NovaFluor Blue 610-70S	Thermo Fisher Scientific	M005T02B06
Anti-Mouse TCRγδ PE-CF594	BD Biosciences	563532
Anti-Mouse NKp46 APC	Thermo Fisher Scientific	17-3351-82
Anti-Mouse CD8 Alexa Fluor 488	Thermo Fisher Scientific	53-0081-82
Anti-Mouse CD49b APC	Thermo Fisher Scientific	17-5971-82
Anti-Mouse CD4 PE-Cy7	Thermo Fisher Scientific	15-0041-82
Anti-Mouse CD8 PE-Cy5	Thermo Fisher Scientific	15-0081-82
Anti-Mouse CD11b Alex Fluor 488	BD Biosciences	567768
Anti-Mouse B220/CD45R V450	BD Biosciences	560473
Anti-Mouse MHCII Alexa Fluor 700	BioLegend	107622
Anti-Mouse CD11c BV570	BioLegend	117331
Anti-Mouse CD127 PE	Thermo Fisher Scientific	12-1271-82
Anti-Mouse EpCAM eFluor 450	Thermo Fisher Scientific	48-5791-82
Anti-Mouse CD49b PE	BD Biosciences	553858
Anti-Mouse CD127 PerCP-Cy5.5	Thermo Fisher Scientific	45-1271-82
Anti-Mouse Sca-1 Alexa Fluor 700	Thermo Fisher Scientific	56-5981-82
Anti-Mouse c-Kit (CD117) APC	BD Biosciences	553356

Supplemental Table 2. Intracellular antibodies used in this project.

Antibody	Manufacturer	Catalog Number
Anti-Mouse IFNγ PE	Thermo Fisher Scientific	12-7311-82
Anti-Mouse IL-13 PE-Cy7	Thermo Fisher Scientific	25-7133-82
Anti-Mouse IL-17A BV605	BD Biosciences	564169
Anti-Mouse Foxp3 Alexa Flour 700	Thermo Fisher Scientific	56-5773-82
